# Combinatory use of distinct single-cell RNA-seq analytical platforms reveals the heterogeneous transcriptome response

**DOI:** 10.1038/s41598-018-21161-y

**Published:** 2018-02-22

**Authors:** Yukie Kashima, Ayako Suzuki, Ying Liu, Masahito Hosokawa, Hiroko Matsunaga, Masataka Shirai, Kohji Arikawa, Sumio Sugano, Takashi Kohno, Haruko Takeyama, Katsuya Tsuchihara, Yutaka Suzuki

**Affiliations:** 1 Department of Computational Biology and Medical Sciences, Graduate School of Frontier Sciences, The University of Tokyo, Kashiwa, Chiba, 277-8562 Japan; 2 Division of Translational Genomics, The Exploratory Oncology Research and Clinical Trial Center, National Cancer Center, Kashiwa, Chiba, 277-8577 Japan; 3 Department of Life Science and Medical Bioscience, Waseda University, Shinjuku-ku, Tokyo, 162-8480 Japan; 4 Hitachi Ltd., Research & Development Group, Kokubunji-shi, Tokyo, 185-8601 Japan; 5 Division of Genome Biology, National Cancer Center Research Institute, Chuo-ku, Tokyo, 104-0045 Japan

## Abstract

Single-cell RNA-seq is a powerful tool for revealing heterogeneity in cancer cells. However, each of the current single-cell RNA-seq platforms has inherent advantages and disadvantages. Here, we show that combining the different single-cell RNA-seq platforms can be an effective approach to obtaining complete information about expression differences and a sufficient cellular population to understand transcriptional heterogeneity in cancers. We demonstrate that it is possible to estimate missing expression information. We further demonstrate that even in the cases where precise information for an individual gene cannot be inferred, the activity of given transcriptional modules can be analyzed. Interestingly, we found that two distinct transcriptional modules, one associated with the Aurora kinase gene and the other with the DUSP gene, are aberrantly regulated in a minor population of cells and may thus contribute to the possible emergence of dormancy or eventual drug resistance within the population.

## Introduction

Recent developments in single-cell sequencing technologies have opened the possibility of analyzing individual single cells. A number of reports have demonstrated that single-cell analysis provides pivotal information for elucidating cellular plasticity and diversity within a given population of cells *in vitro* and *in vivo*. There are a number of potential applications for scRNA-seq analysis. Among them, cancer is supposed to be one of the most important targets to analyze^1^. Cancer is a complex cellular ecosystem that consists of various cell types, including cancer cells, cancer-associated fibroblasts, tumor-infiltrating leukocytes and vascular cells^2^. In cancer, no two individual cells are identical, as they are located in varying micro-environmental conditions and interact with different cells. Even among clonal cancer cells, diverse phenotypic features are frequently observed in each individual cell during the clonal evolution of cancers^3–5^. It is important to focus on the molecular diversity of cancer cells to understand the mechanisms underlying the emergence of drug-resistant and metastasizing cells. Detailed knowledge of such intra-tumor heterogeneity would provide crucial information for understanding the eventual development of drug-resistant cells or metastatic dissemination in cancer and also generate potential opportunities for novel pharmaceutical interventions^6,7^. Most cancers acquire resistance to anti-cancer drugs, including gefitinib, one of the most well-characterized molecular-targeting anti-cancer drugs for EGFR in lung adenocarcinomas, after a period of drug treatment^8–10^. Several drug resistance-acquiring mutations, such as the T790M mutation in the EGFR gene, have been reported^11^. These drug-resistant mutations emerge in a small number of cells and spread to the population^12^. Before drug resistance is fixed in the form of genomic mutations, transcriptomic diversity is considered to be the precedent for the initial repertoire, allowing cells to survive during initial selection^13^. Details of the molecular process that eventually leads to drug resistance remain mostly elusive despite several pioneering studies^14^. It has been difficult to examine cellular diversity using conventional methods, which subject groups of cells to assays in bulk. Limited amounts of data represent cancer cell heterogeneity, even with the most powerful datasets describing cancers, such as the TCGA, ICGC and COSMIC databases^15–19^.

It is expected that single-cell analysis will provide substantial novel insights into the identification and characterization of rare cells among cancer cells^20–24^. Such single-cell analyses were initiated with single-cell RNA-seq (scRNA-seq) analysis. In scRNA-seq, single cells are separated by micro-pipetting, laser capture micro-dissection, FACS, micro-fluidic and micro-droplet-based methods^25–29^. Several fully- or semi-automated platforms have become commercially available for separation, facilitating scRNA-seq and robust analytical methods^30^. From a technical perspective, these methods can be separated into two categories. In the first category, cells are physically separated by microfluidics, FACS, or other methods, followed by the application of, for example, reverse transcription, cDNA amplification and adaptor tagging in an individual reaction chamber manually or with the aid of robotics (“micro/nano-chamber” platform)^31,32^. In the second category, cells are confined to a micro-droplet, and individual cells are separately marked using molecular barcoding technology (“micro-droplet” platform)^33,34^. The commercial platforms representing these technologies are the C1 system of Fluidigm and the Chromium system of 10× Genomics, respectively^32,35^. Although both platforms are commonly utilized for scRNA-seq, each has several intrinsic advantages and disadvantages. For a given sequencing project and human costs, the micro-chamber-based method yields sufficient sequence coverage per cell but sparse coverage of the cellular population. Design of the micro-chamber based methods has obvious physical limitations and thus cannot be scaled to thousands of cells. On the other hand, the micro-droplet-based methods yield sufficient coverage of cellular populations but sparse coverage per cell. We consider neither method on its own to be sufficient to provide a full spectrum of information for the single-cell transcriptome, and mutual complementation should thus be necessary. However, to our knowledge, no study has demonstrated how such integration can be implemented. First, no precise evaluation of the platforms has been reported to address the extent of data consistency between the two methods.

In this study, we attempted to combine the scRNA-seq datasetes obtained from the micro-chamber and micro-droplet platforms. In particular, we focused on transcriptomic heterogeneity in response to an anti-cancer drug in lung adenocarcinoma-derived cell lines. We used five cell lines, PC9, II-18, H1975, H1650 and H2228. PC9 and II-18 cells harbor driver mutations in the EGFR gene and a 15-base-pair-deletion in exon 19 and L858R, respectively. These cell lines are thus sensitive to gefitinib. H1975 cells carry the L858R mutation as well as a secondary mutation at T790M, resulting in complete resistance to the first-generation EGFR tyrosine kinase inhibitor. H1650 cells have a deletion in exon 19 of EGFR and PTEN gene loss, and they thus exhibit partial resistance^36,37^. H2228 cells have an ALK-fusion as a driver mutation, so gefitinib should be ineffective^38,39^. Using these cell lines, we collected scRNA-seq data from a total of 536 and 54,631 single cells using a micro-chamber-based platform, bead-seq, and a micro-droplet-based platform, Chromium of 10× Genomics, respectively. Readers are directed to the Supplementary information (Sup. Fig. S1) a comparison of bead-seq and the commercial method C1. In the first part of this paper, we primarily focus on the technical evaluation of the scRNA-seq data obtained from the respective platforms. Using the scRNA-seq data obtained from PC9 cells, we evaluate the robustness of the data and concordance between the two platforms. In the next part of the study, we attempt to infer the missing values for the micro-droplet dataset by statistical inference from the micro-chamber dataset. We further attempt to determine the cellular heterogeneity of a given population of cells by utilizing the co-expression network analysis-based module identification method. We also describe our attempts to elucidate the heterogeneous transcriptomic responses of cancer cells in response to an anti-cancer drug.

## Results and Discussion

### scRNA-seq analysis of single cells of a series of lung adenocarcinoma cell lines

We constructed and analyzed a series of single-cell RNA-seq libraries using five human lung adenocarcinoma-derived cell lines (Table 1). The cells were stimulated with or without an EGFR-targeting anti-cancer drug, gefitinib (see the Materials and Methods for the stimulation conditions and cellular responses). We constructed the libraries from the same materials using both a micro-chamber-based platform, bead-seq, and a commercial micro-droplet-based platform, Chromium of 10× Genomics^31,35^. We followed the standard experimental and analytical procedures for each platform. For the micro-chamber datasets, sequencing was conducted by 50-base single-end reads. For 47 cells of each sample (22 cells only for PC9 cells treated with gefitinib), an average of 1.6 million reads per cell was obtained. Of the mapped reads, 87% overlapped with the RefSeq regions. Expression levels were calculated for each gene as reads per kilobase RNA per million mapped tags (RPKM) (Table 2, top). For the micro-droplet datasets, sequencing was conducted by 50-base paired-end reads. For all the samples, approximately 100–170 million sequence reads were generated per library. After removing PCR duplicates and separating the scRNA-seq tags for individual cells based on unique molecular identifiers (UMIs), 83,649,012 reads remained on average (57%, Sup. Table S1). We further removed the data representing cells with only small numbers of scRNA-seq tags. As a result, we obtained scRNA-seq data for 3,000–5,000 cells with more than 5,000 RNA-seq tags. The sequence data were similarly associated with the RefSeq genes, and the expression levels were calculated as parts per million mapped tags (ppm) for each gene (note that the micro-droplet method is the 3′-end sequencing method) (Table 2, bottom).

**Table 1:** Cell-type data used for the present study.

Cell line	Response to gefitinib	Mutation
PC9	sensitive	EGFR Exon19 del
II-18	sensitive	EGFR L858R
H1650	resistant	EGFR Exon 19del, PTEN loss
H1975	resistant	EGFR L858R, T790M
H2228	resistant	EML4-ALK fusion gene

**Table 2:** Statistics of the two platform datasets used in the present study.

**Statistics of the micro-chamber system data**				
Cell line	Treatment	Average raw reads	Average used reads	Number of cells
PC9	DMSO	1,938,496	1,683,528	44
	gefitinib	1,728,426	1,502,134	22
II-18	DMSO	1,071,312	904,648	47
	gefitinib	1,169,322	985,941	47
H1650	DMSO	1,158,863	1,018,527	47
	gefitinib	1,283,908	1,126,651	47
H1975	DMSO	846,215	725,523	47
	gefitinib	866,011	744,728	47
H2228	DMSO	1,362,097	1,183,384	47
	gefitinib	1,269,704	1,092,511	47
PC9 replicate	DMSO	1,362,097	1,183,384	47
Statistics of the micro-droplet system data used in the present study				
**Cell line**	**Treatment**	**Total raw reads**	**Total used reads**	**Number of Cells with >5 k tags**
PC9	DMSO	127,631,437	64,433,148	5,166
	gefitinib	170,913,768	55,473,258	4,378
II-18	DMSO	173,605,651	57,839,961	4,965
	gefitinib	164,734,523	65,419,730	5,287
H1650	DMSO	100,094,271	49,223,558	4,348
	gefitinib	111,008,829	54,577,170	5,140
H1975	DMSO	161,777,133	33,499,006	3,079
	gefitinib	173,096,952	56,825,197	4,940
H2228	DMSO	176,666,104	61,861,660	5,354
	gefitinib	149,011,834	43,740,708	5,008
H1975 replicate	DMSO	139,741,411	59,100,428	5,152

### Technical evaluations of the two scRNA-seq platforms

Using the obtained datasets, we conducted a series of technical evaluations. Tentatively, only the results from the data from PC9 cells are shown. The results were also obtained from other datasets and are summarized in Supplementary Fig. 2 (Sup. Fig. S2A–E).

**Figure 1:**
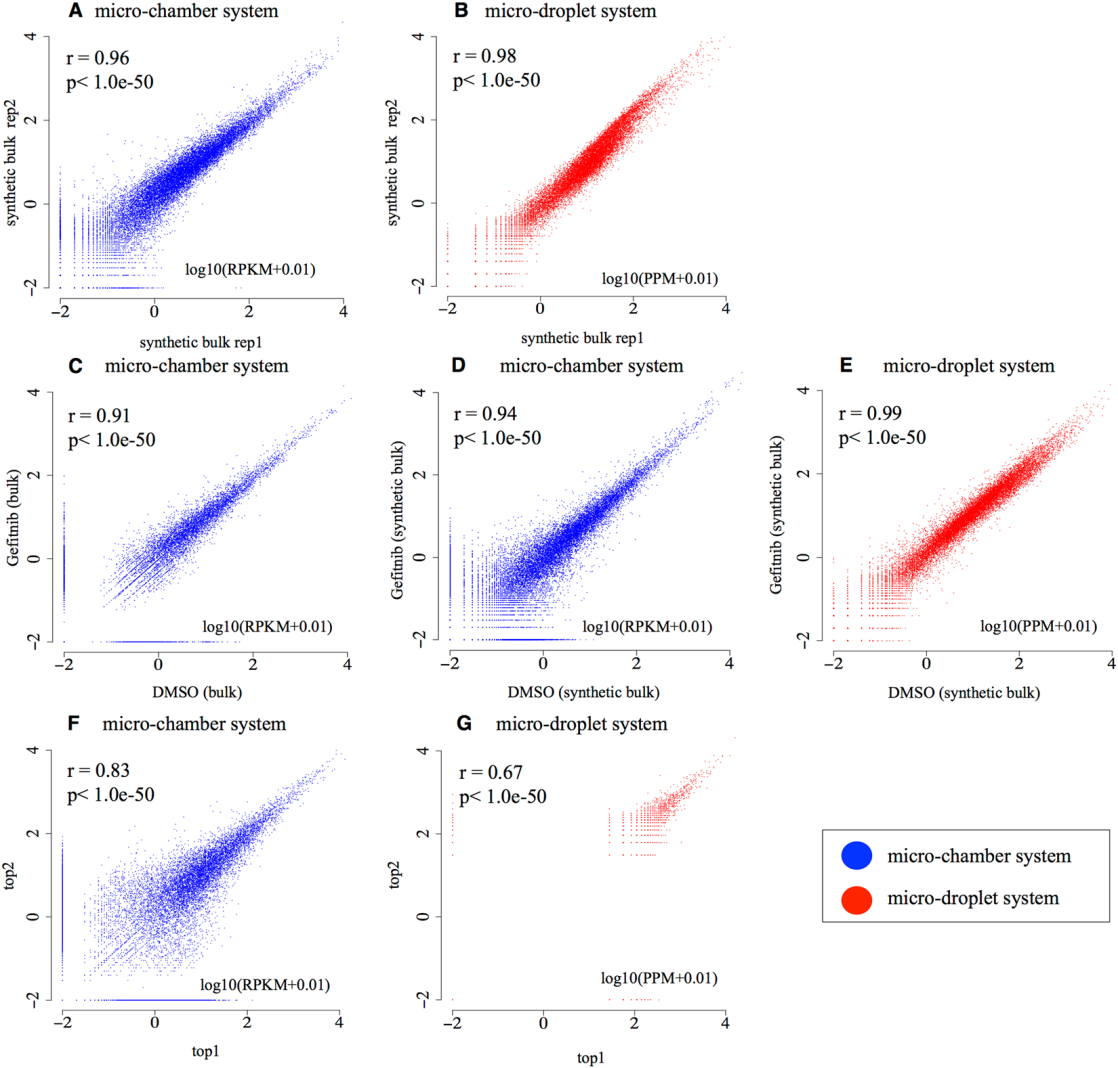
Generation of the RNA-Seq dataset using the micro-chamber system and the micro-droplet system. (**A**,**B**) Comparison between biological duplicates of PC9 cells in the micro-chamber system (**A**) and H1975 cells in the micro-droplet system (**B**). The Pearson’s correlation coefficient of the two experiments is shown in the plot. (**C**) Gene expression changes in response to gefitinib treatment in the PC9 bulk sample in the micro-chamber system. The Pearson’s correlation coefficient of the two experiments is shown in the plot. (**D**,**E**) Gene expression changes in response to gefitinib treatment in the PC9 synthetic bulk sample in the micro-chamber system (**D**) and in the micro-droplet system (**E**). The correlation between the two experiments and the p-values for the Pearson’s correlation are shown in the plot. (**F**,**G**) Validation of PC9 cell expression levels in the top1 and top2 cells. “top1” and “top2” cells are the cells having the largest and the second largest number of sequence tags, respectively, for each of the platforms. “top1” and “top2” cells had the sequence tags of 2,944,039 and 2,773,525 tags in the micro-chamber system (F) and 35,753 and 32,472 in the micro-droplet system (G), respectively. The correlation between the two experiments and the p-value from the Pearson’s correlation are shown in the plot.

First, we evaluated the reproducibility of each platform at the level of the “synthetic bulk,” which represents the average of the scRNA-seq data for a given population of cells. We observed a strong Pearson’s correlation between independent experiments for both platforms (r = 0.96 and r = 0.98; Fig. 1A and B). We also examined the scattering plots, comparing the expression patterns between untreated and gefitinib-treated cells, and found that the results were consistent with those obtained from the analysis of bulk cells under similar conditions (Pearson’s r = 0.91 for the bulk dataset with a micro-chamber system; r = 0.94 for the synthetic bulk dataset with a micro-chamber system; r = 0.99 for the micro-droplet system synthetic bulk dataset; Fig. 1C–E). Second, we evaluated the scRNA-seq data at the single-cell level. We selected two cells, one with the largest and one with the second largest number of mapped scRNA-seq tags (the top1 and top2 cells), from the each platform. In the micro-chamber dataset, the top1 and top2 cells had 2,944,039 and 2,773,525 tags, while those in the micro-droplet datasets had 35,753 and 32,472 tags, respectively. We compared the observed gene expression levels in the top1 and top2 cells for each method. Correlations in expression information were reasonable for both platforms (Pearson’s r = 0.83 and r = 0.67; Fig. 1F and G), even though the correlation was less significant for the micro-droplet platform (for further discussion, see below). These results collectively suggest that data from both platforms are highly reproducible and robust when evaluated from these perspectives.

### Distinct features of the different platforms

**Figure 2:**
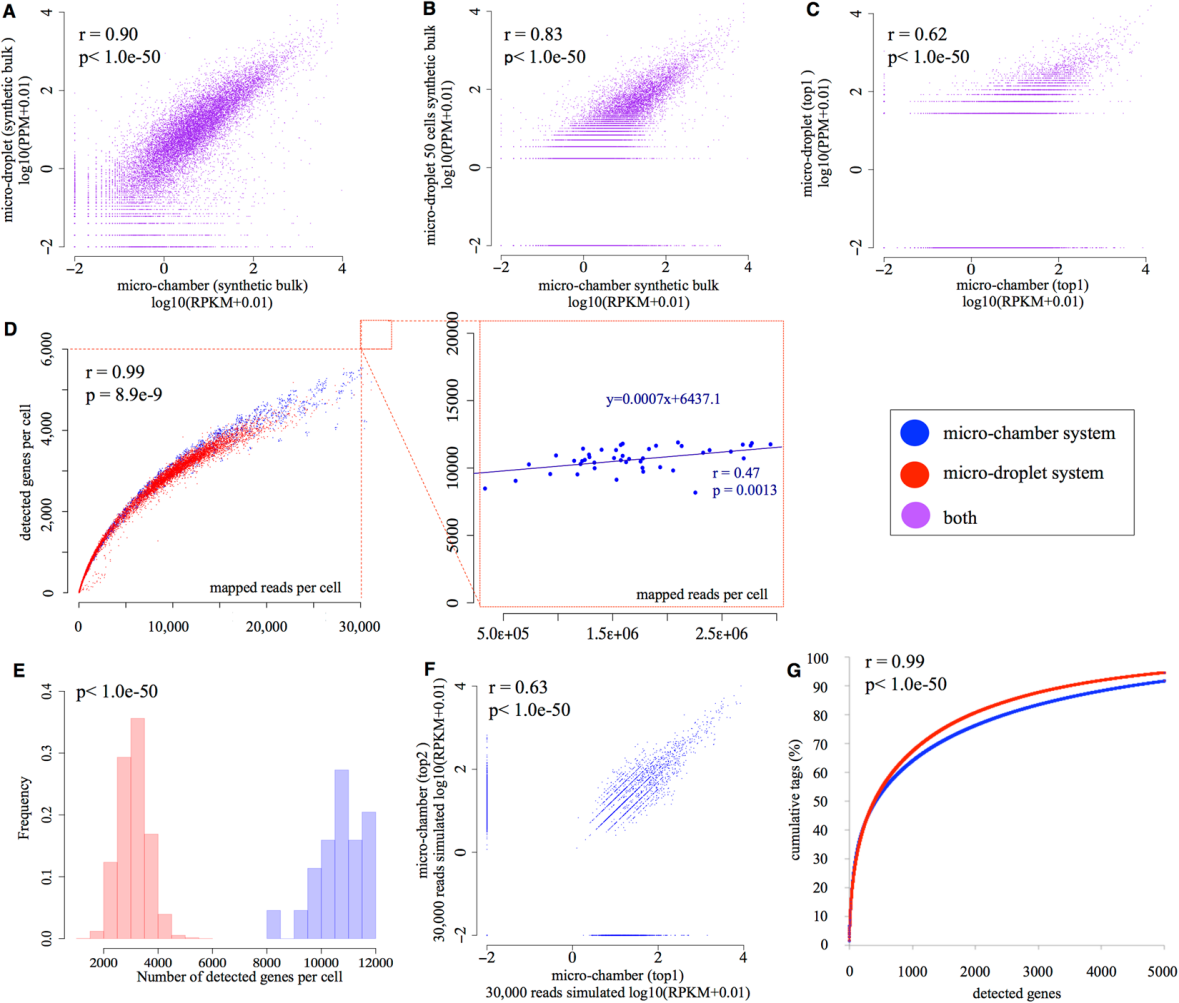
Diversity in the expression levels between different individual cells and different genes. (**A**–**C**) Comparison between synthetic bulk expression in the micro-chamber (*x*-axis) and the micro-droplet systems (*y*-axis) (**A**), between the micro-chamber (*x*-axis) and the micro-droplet systems at the virtual bulk level. For the micro-droplet dataset, the cells were computationally diluted to 50 cells (*y*-axis) (**B**). Results of the similar analysis between the top1 cell from each system. The cell having the largest and the second largest number of mapped tags are designated as the top1 and top2 cells, respectively for each of the platforms (**C**). The correlation between the two experiments and the p-values of the Pearson’s correlation are shown in the plot. (**D**) Correlation between the number of mapped reads per cell and the number of detected genes per cell in the micro-chamber system (simulated) and the micro-droplet system (left) and the micro-chamber system (experimental, right). To evaluate the statistical significance in the difference between the distribution patterns of red (micro-droplet) and blue (micro-chamber) dots, 1,500 cells were randomly selected from micro-chamber simulated data and micro-droplet data, Correlation between two platforms were evaluated as the p-values of the Pearson’s correlation are shown in the plot (left). In the right panel, the regression line and the Spearman’s correlation and the p-values are also written in the plot. (**E**) Correlation between the number of detected genes per cell (*x*-axis) and the frequency (*y*-axis). The p-value of the Wilcoxon’s rank sum test is shown in the plot. (**F**) Comparison between two micro-chamber samples simulated to have 30,000 tags per cell. The correlation between the two samples and the p-values of the Pearson’s correlation are shown in the plot. (**G**) Comparison between the number of detected genes (*x*-axis) and cumulative number of tags (*y*-axis) in the two systems.

We next compared the datasets obtained from the two platforms. We observed a generally high correlation between them when the results were evaluated at the synthetic bulk level (Pearson’s r = 0.90; Fig. 2A), ensuring concordance between the platforms and again confirming the robustness of both platforms. Particularly, to compare the features of the datasets produced from the two different platforms, we computationally generated the data for 50 cells from the micro-droplet datasets. Then, we compared them with that of the micro-chamber datasets. Even when compared the datasets using the same number of the cells, we still found the degree of the correlation was comparable to that of Fig. 2A, thus, the difference in the number of the analyzed cells may not be the main causing factor (Fig. 2B, r = 0.83). Also, we compared the datasets between the platforms at the single-cell level using the top1 cell from each dataset, a remarkable decrease in correlation was observed (Pearson’s r = 0.62; Fig. 2C). To determine the generality of this observation, we conducted a similar analysis using other cells, namely, the top1, top2, and top 3 cells (the cells having the highest number of sequence tags and the second and third highest, respectively) from each datasets. The results were essentially the same when we used other cells for the same analysis (r = 0.55–0.63, Sup. Fig. S3). Even though this analysis did not cover all the cells included in the respective datasets, the obtained results raised substantial concern that there might be a discrepancy between the two platforms observed at the single cell level. To further address this issue, we inspected the intrinsic features of the datasets. We first examined the number of genes represented per single cell in the each dataset. Not surprisingly, the number of represented genes increased as a proportion of the increasing sequence depth (Fig. 2D, left). At a given sequence depth, an average of 10,661 and 3,096 genes were represented per cell in the chamber and micro-droplet platforms, respectively (Fig. 2E). When we computationally separated the datasets and simulated the dependency of the number of detected genes on the sequence depth, we found similar curves with increasing trends for the micro-chamber and the micro-droplet datasets, suggesting that the data obtained by the two platforms had highly similar properties in this regard (Fig. 2D, left). In addition, we randomly selected 30,000 reads, representing slightly fewer reads than found in the top1 and top2 samples in the micro-droplet dataset, from the two micro-chamber datasets and drew a scatterplot. The result revealed a correlation similar to that in the micro-droplet dataset (Fig. 1G, r = 0.67, <1.0e–50, Fig. 2F, r = 0.63, p < 1.0e–50). On the other hand, we also reduced the number of the sequence tags per cells to normalize the sequencing depth between the two platforms. Again, we obtained the similar results, even when the numbers of the sequence tags per cells were the same between the two platforms (Fig. 2G, r = 0.99, p < 1.0e–50). While the number of detected genes, depending on the sequence depth, had almost reached a plateau for the micro-chamber dataset (the inclination rate of the regression line was 0.0007; Fig. 2D, right), the micro-droplet dataset was still in a long-phase inclination. These results collectively indicated that the nature of the data from the two platforms was essentially the same. However, the representation of the data, which had been derived from a random selection of a given number of cells from the entire population, differed depending on the sequence depth. As a result, the datasets from the two platforms were highly concordant when compared at the synthetic bulk level, where the sequence depth is sufficient, and the discrepancy became apparent when the datasets were evaluated at the single-cell level. These results also indicated the possibility that the two datasets could be used for the integrative data analysis in a mutually complementary manner, since they do not have obvious different features.

### Distinct expression information from each platform for individual genes

**Figure 3:**
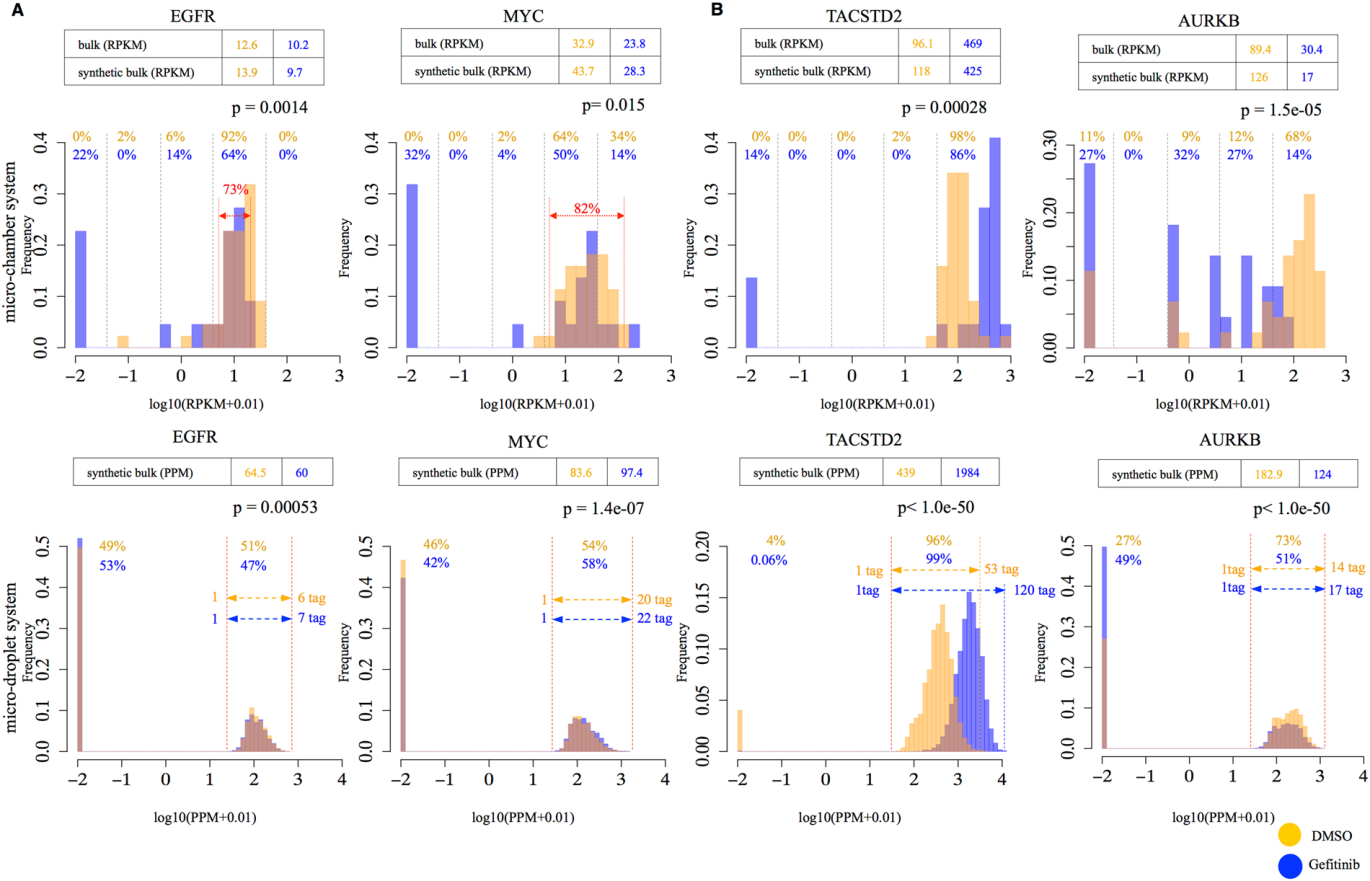
Information about individual gene expression of the platforms. (**A**,**B**) The relationship between the expression levels and the distribution of representative cancer-related genes, EGFR (left) and MYC (right) (**A**), and TACSTED2 (left) and AURKB (right) (**B**), using both platforms. The dashed red line in Fig. 2A shows average ± SD rpkm for each gene (13.7 ± 7.6 and 36.4 ± 31.8 rpkm).

We further scrutinized the gene expression levels and their changes in response to the drug treatment as determined for representative genes by the different platforms (Fig. 3A and B). For example, the expression levels were at ~10 and ~30 RPKM at the bulk RNA-seq level for the EGFR and the MYC genes, respectively. We report the expression in most of the samples under untreated conditions in the micro-chamber dataset as the average ± SD RPKM (Fig. 3A top, within dashed red line area, 72.7% for EGFR and 81.8% for MYC among the existing untreated samples). After gefitinib treatment, the expression of these genes in some populations of cells was no longer detected, perhaps due to the termination of the EGFR pathway signal. In contrast, when we looked at the same expression changes using the micro-droplet dataset, we found that some cells had a gene expression level of 0 under untreated conditions simply due to the lack of any scRNA-seq tags for those cells. The levels in cells with observable expression levels were represented by only a small number of tags (see the insets, Fig. 3A, bottom). Changes in expression patterns in response to the drug, as represented in the micro-chamber dataset, were lost in the micro-droplet dataset.

When we examined other genes, we roughly estimated that the observed expression levels should be reasonably reliable for genes with expression levels exceeding approximately 500 RPKM. Below that expression level, the gene expression information may not be precisely represented or expression of the genes themselves may not be detected, depending on their expression level. The TACSTD2 gene, which is a potential tumor suppressor gene in lung cancers^40,41^, is a typical example (Fig. 3B, left). When the expression level of this gene was 439 ppm (and 1,984 ppm in the synthetic bulk), precise expression information, variance between cells and fold changes in response to the drug treatment were precisely represented (Fig. 3B, left, bottom). The case of the Aurora Kinase B gene, which is a kinase of the pivotal cell cycle regulatory protein p53 and is frequently associated with carcinogenesis in various cancers, is shown in a panel^42^ (Fig. 3B, right). When we examined the micro-chamber dataset for the expression of this gene, we observed that its expression varied between individual cells, perhaps depending on the cell cycle of the corresponding cells. However, such important variation in the expression patterns of this gene between cells was not represented in the micro-droplet dataset due to the discrete counts of RNA-seq tags at this expression level. It is also noteworthy that the fold changes observed in gene expression in response to gefitinib at bulk levels varied between genes depending on their expression levels (Table 3).

**Table 3:** Comparison of the expression levels of representative genes and their changes. Expression levels of four genes in the bulk and synthetic bulk samples of both platforms.

			EGFR	MYC	TACSTD2	*AURKB
micro-chamber	bulk (RPKM)	treated	10.18	12.6	468.68	30.35
		untreated	12.6	32.86	96.1	89.43
		fold (untreated/treated)	1.23	2.61	0.21	2.95
	synthetic bulk (RPKM)	treated	9.69	28.31	424.69	17.2
		untreated	13.87	34.68	118.34	126.39
		fold (untreated/treated)	1.43	1.23	0.28	7.35
micro-droplet	synthetic bulk (PPM)	treated	59.9	97.4	1985	124.25
		untreated	64.45	83.6	439.47	182.9
		fold (untreated/treated)	1.08	0.86	0.22	1.47

*Difference is most significant.

**Figure 4:**
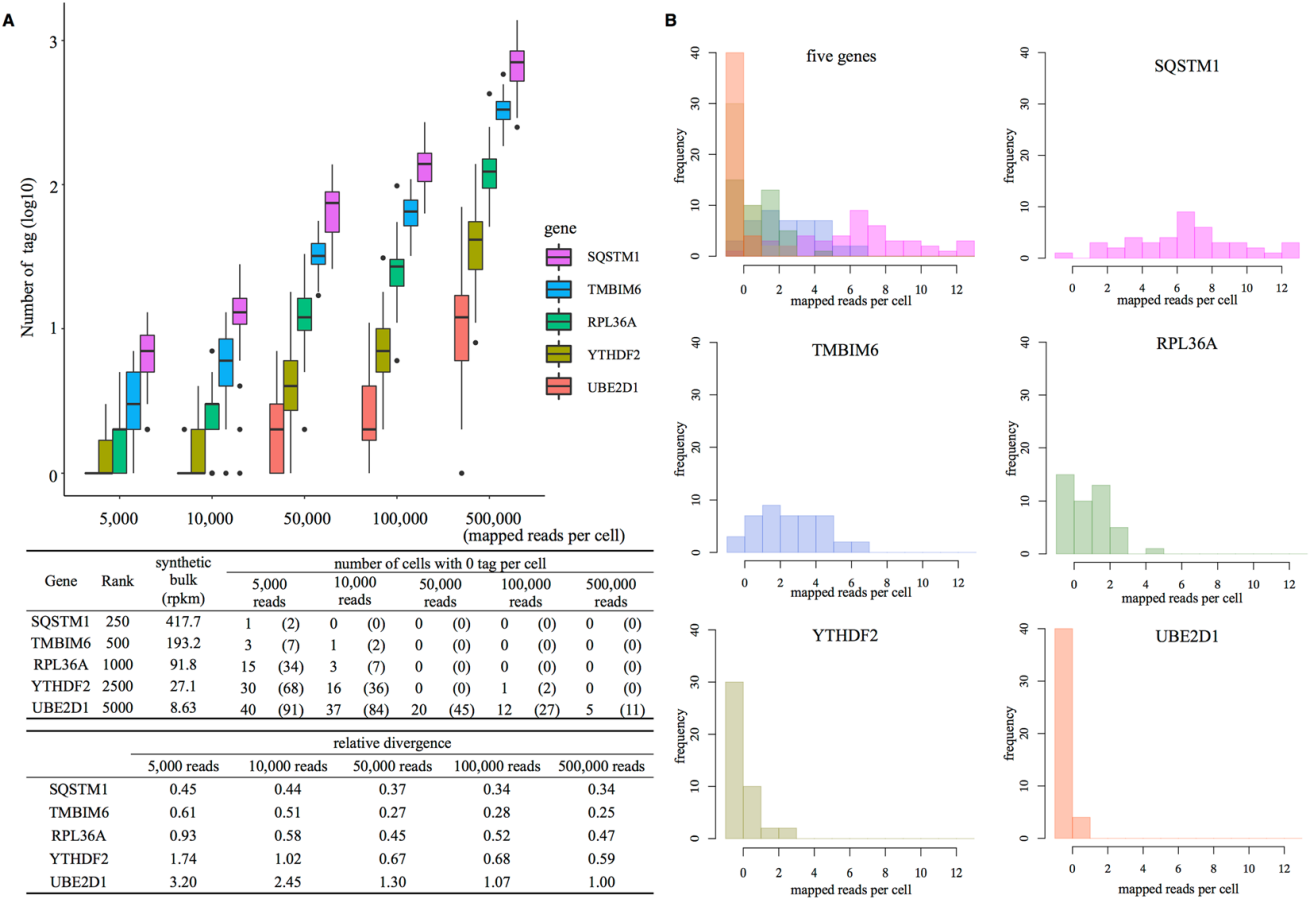
Number of the sequence tags representing the gene expression information. (**A**) Five genes at varying average expression levels at bulk were selected and the number of the sequence tags representing their expression levels (y-axis) at the indicated sequence depth (*x-*axis) is shown. Sequence tags per cell were serially diluted computationally starting from the micro-chamber datasets. Error bars correspond to the divergence between individual cells. Since, the cells having zero tags are not represented explicitly, those populations are separately indicated in the table in the margin. Cellular divergences of the number of the sequence tags are also represented in the most bottom table. (**B**) Results of the similar analysis are shown in separately for individual genes at the average sequencing depth of 5,000 tags per cell.

Indeed, we examined and found that the gene expression information is not always correctly represented particularly for the genes having low expression levels at the shallow sequencing depth (Fig. 4A). For example, in the case of the UBE2D1 gene (Fig. 4B, bottom right panel), many of the cells had the zero tag count to represent its expression levels at the average sequencing depth of 5,000 tags per cell Computational serial dilution of the sequence tags per cell indicated at least 100,000 tags per cell was needed to correctly represent the gene expression levels and cellular divergence of its gene expression. The bottom table of Fig. 4 summarizes the results of the similar analysis for other genes. Again, these results indicated that the datasets obtained from the two platforms have the similar nature.

### Cellular status represented in the different scRNA-seq datasets

**Figure 5:**
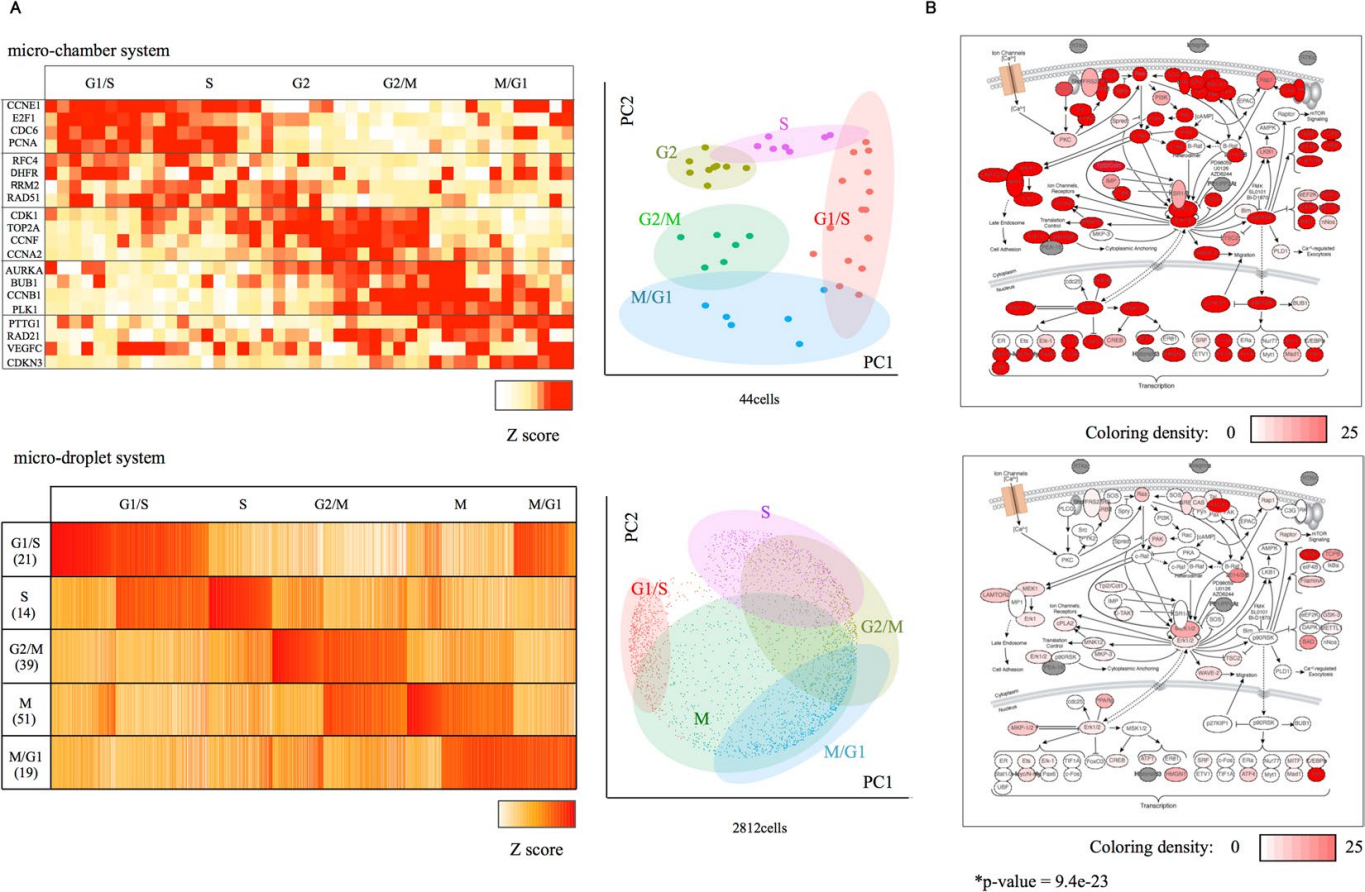
Cell cycle Analysis and Gene Expression information. (**A**) Cell cycle analysis of PC9 cells from the micro-chamber system datasets (top) and micro-droplet system datasets (bottom). A heatmap (left) and PCA analysis (right) are shown. (**B**) Gene expression information mapped to the MAPK pathway. The color density of the pathway represents the expression levels of the top1 cell from the micro-chamber system (top) and micro-droplet systems (bottom). The p-value of the Wilcoxon’s rank sum test is shown in the plot.

We also examined how biological information was represented in the respective datasets. First, we examined cell cycle-dependent gene expression (Sup. Table S2). We particularly attempted to determine the cell stage of the corresponding cells. Based on the heat map shown in Fig. 5A, we could estimate the cell cycle states for most of the cells based on the micro-chamber dataset (Fig. 5A, left). Starting from the micro-droplet dataset, essentially similar maps could be drawn even though the map was somewhat blurred when it was focused on each cell cycle (Fig. 5A right, Sup. Table S3 and Fig. S4). It is noteworthy that those genes that were used for cell cycle separation generally had relatively high expression levels.

We further examined how the gene expression levels for a particular signaling pathway were represented for a given single cell. As exemplified for the MAPK pathway, which is a pivotal pathway in lung cancers, reasonable expression information was represented by the micro-chamber dataset for the top1 cell (Fig. 5B, top). However, almost no gene expression information was available in the micro-droplet dataset, even for the top1 cell (Fig. 5B, bottom). Taken together, we concluded that, although the two platforms are based on substantially distinct characteristic principles, the produced data have the similar nature. The difference in the sequencing depth was the only major causing factor for the appeared differences. The effect of this factor is emphasized when the genes of low expression levels are concerned. Indeed, when observed at the single-cell level, the micro-droplet method can detect only a relatively poor representation of the gene expression in general. However, a large number of cells can be analyzed by this method. Vice versa is the case for the micro-chamber method. We therefore considered that an integrated method should be helpful, taking advantages of the generally high concordance between these datasets. We considered it highly beneficial if more precise gene expression information in the micro-droplet dataset, containing information for >5,000 cells, could be inferred at greater precision based on the reliable expression information for each single cell from the micro-chamber dataset, whose expression information represents only <100 cells.

### Estimation of the missing values in the micro-droplet dataset using the micro-chamber dataset

To complement the relative lack of expression information in the micro-droplet dataset, we first assessed the possibility of estimating the missing values of the gene expression levels in this dataset by statistical inference from the micro-chamber dataset. First, we combined the gene expression data from all the micro-chamber RNA-seq datasets shown in Table 1. Based on the obtained datasets, which include result for 442 cells, we selected genes that exhibited an average RPKM >10 in all datasets. We used the gene expression information for the selected genes as predictors to estimate the genes of interest in the micro-droplet dataset. For statistical inference, the expression levels of the genes of interest were predicted by a linear combination of the predictor genes. The predictor genes were selected by variable selection using the LASSO regression to avoid overfitting (for details, see Materials and Methods). The expression levels of all the genes belonging to this pathway were predicted and evaluated by considering Pearson’s correlation between the predicted values and experimentally observed values.

**Figure 6:**
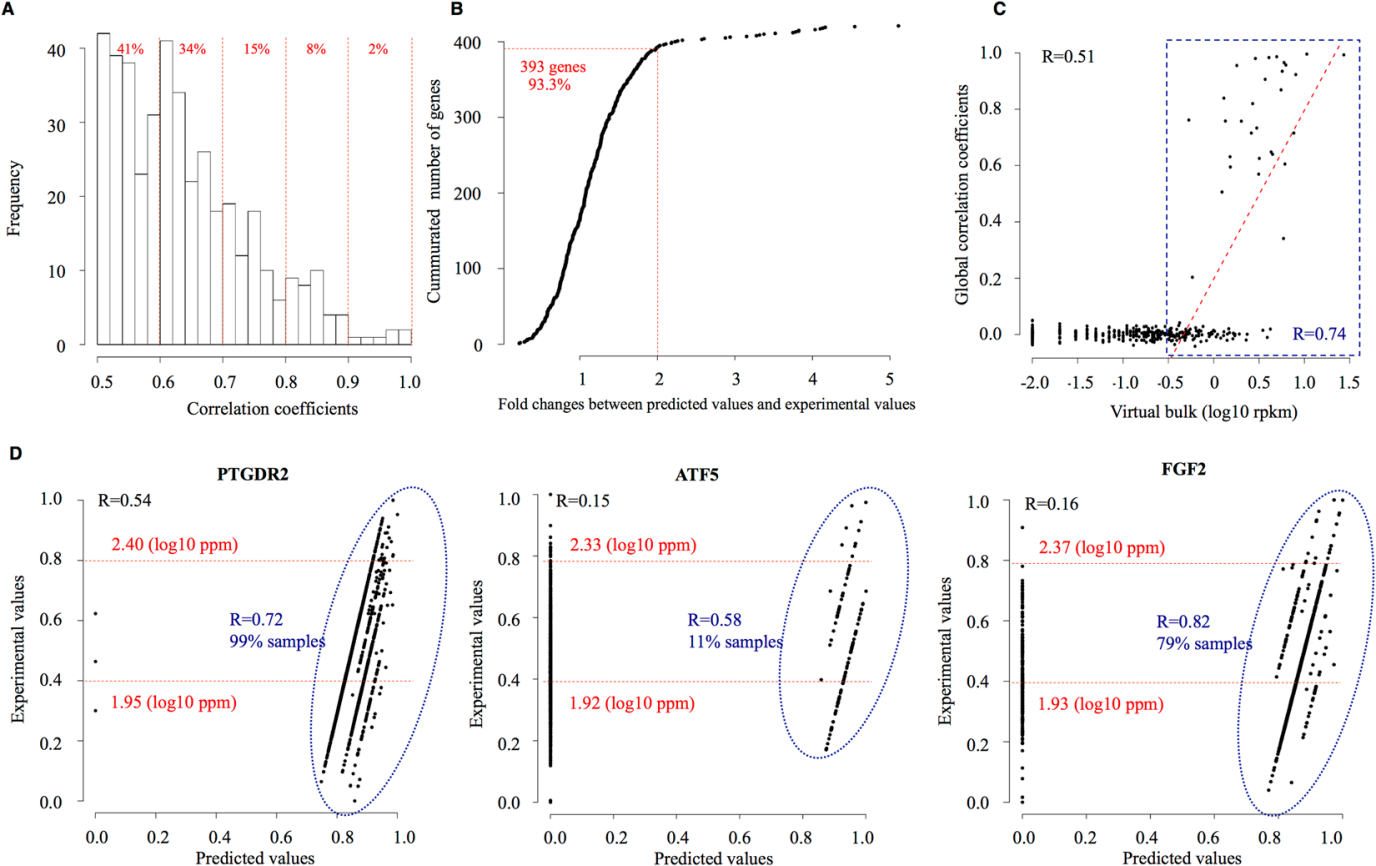
Estimation of values absent from the micro-droplet system dataset by the micro-chamber system dataset. (**A**) Distribution of predictive precision. The correlation coefficients (*x*-axis) and the number of genes (*y*-axis) are shown. The gene distribution for each correlation coefficient is shown in the upper margin of the plot. (**B**) Fold differences between predicted and experimental values (*x*-axis) and the cumulated number of genes (*y*-axis). (**C**) Prediction validation. Correlation between expression levels (*x*-axis) and global correlation coefficients (*y*-axis). Genes with R > 0.5 and number of validated samples >5 are shown (421 genes). A fitting line was plotted for genes with virtual bulk expression levels >−0.5 (log10 RPKM), and a correlation coefficient of 0.74 was obtained for these genes. (**D**) Comparison between normalized predicted values (*x*-axis) and normalized experimental values (*y*-axis) of three genes. We used 0-1 normalization for the different scales. The Pearson’s correlation coefficients comparing values of all cells (black) and selected cells (blue) are shown in the plot. All experimental values shown in the plots were obtained from DMSO-treated experiments. Experimental expression levels (log10 PPM) corresponding to 0.8 and 0.4 are also shown in the plot (red).

As a result, we were able to infer the gene expression levels in a reasonably precise manner for many genes. Predicted and observed values correlated at a level of r > 0.5 for 421 of the genes (Fig. 6A). The missing values of the gene expression levels for 393 genes were inferred within two-fold for 93.3% of the cases (Fig. 6B). When we further scrutinized the data, we found that accuracy of the inference depended on the levels of the target genes and the predictor genes (Fig. 6C). When the expression levels of relatively highly expressed genes were predicted (as exemplified by the PTGDR2 genes; 7.61 RPKM in the micro-chamber bulk, Fig. 6D left), the inference was successful for the majority of cells in a highly accurate manner. When the expression levels were normal (as exemplified by the ATF5 gene; middle panel, 5.7 RPKM in the micro-chamber bulk, Fig. 6D middle) and lower (as exemplified by the FGF2, 1.47 RPKM in the micro-chamber bulk, Fig. 6D right), the inference was gradually affected by both the number of cells and the prediction accuracy. It was similarly difficult to estimate the missing values for genes in the MAPK/ERK pathway due to the missing values of predictors in the corresponding cells (Sup. Fig. S5). Expression levels of seven genes in the MAPK/ERK pathway were estimated (r > = 0.39) in some cells with non-missing values for the predictor genes (Table 4)^43^.

**Table 4:** Estimation of MAPK pathway gene expression levels.

MAPK gene	Predictive precisions	Partially predictive precisions	Proportion of samples
PAK6	−0.12	0.7	30%
SPRED2	−0.16	0.69	3%
MKNK2	−0.03	0.59	16%
MAP2K1	0.03	0.49	3%
EEF2K	−0.22	0.4	53%
ETS1	−0.19	0.39	42%
IRS1	0.06	0.47	16%

### scRNA-seq analysis at the level of regulatory module expression

Next, we considered whether we could analyze the expression information as a particular transcriptome module that consists of mutually correlated genes, even when the expression information for an individual gene itself could not be precisely measured or inferred. To evaluate this possibility, we utilized co-expression networking analysis. We constructed co-expression network modules by applying weighted gene co-expression network analysis (WGCNA; see Materials and Methods for details) for 66 PC9 cells using the micro-chamber dataset (44 cells treated with DMSO and 22 cells treated with gefitinib). We removed five cells with exceptionally low tag counts and selected a total of 13,619 genes with expression levels exceeding 5 rpkm in at least one cell. As a result, we constructed 71 modules (Sup. Fig. S6A). For each of the modules, we considered the module eigengene (ME) to represent the activity of the module^44^.

**Figure 7:**
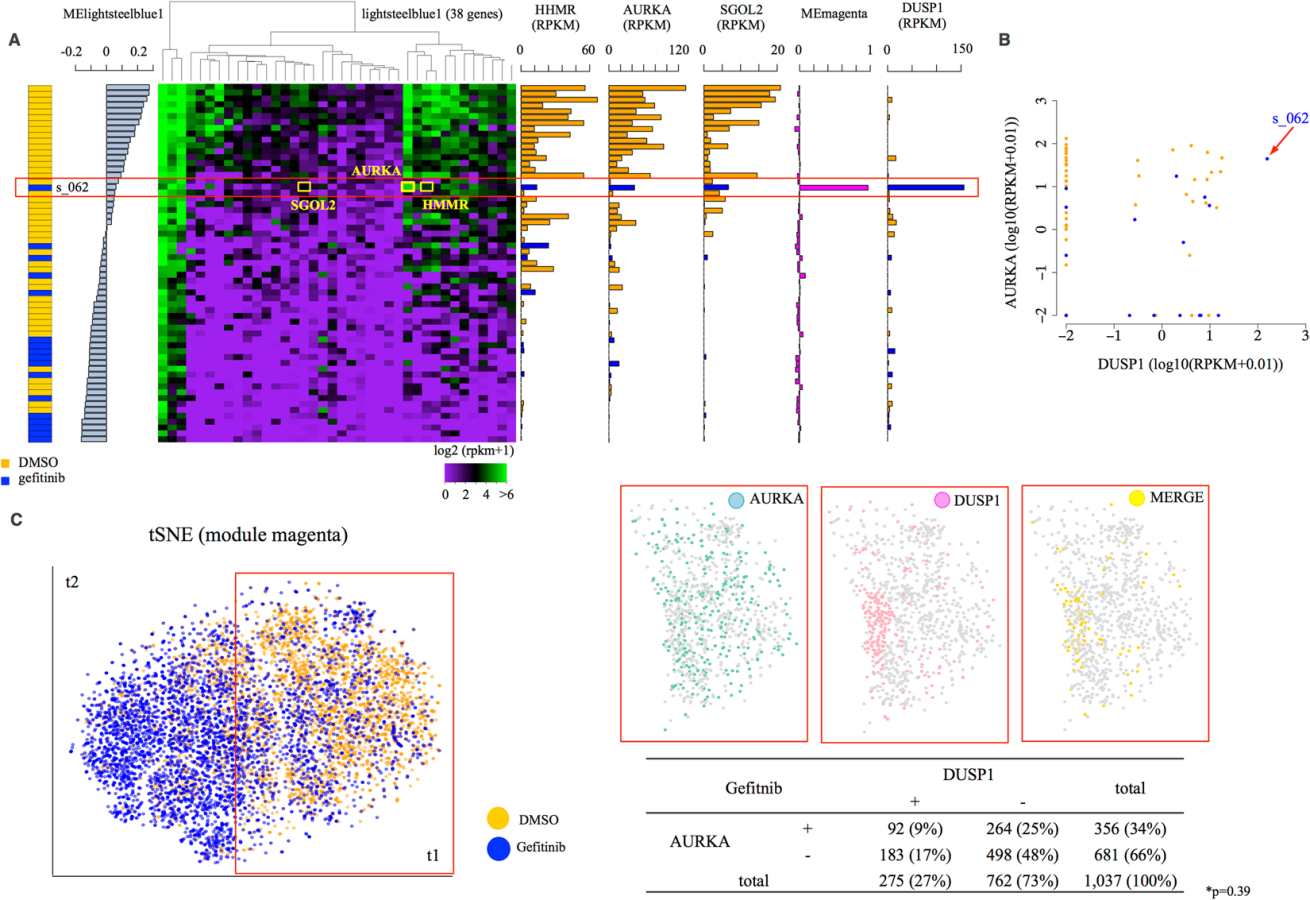
Comparison of the micro-droplet and the micro-chamber systems. (**A**) Hierarchical clustering analyses were conducted using the genes included in the module “lightsteelblue1.” Treatments of individual cells and MElightsteelblue1 values are represented in the left margin of the heatmaps. The expression level of the top3 module genes, MEmagenta values and the expression levels of DUSP1 are represented in the right margin of the heatmap. (**B**) The expression levels of DUSP1 (*x*-axis) and AURKA (*y*-axis) in the micro-chamber dataset samples. (**C**) Clustering of 9,544 PC9 single-cell expression profiles into two cell populations. The plot shows a two-dimensional representation (tSNE) based on the gene expression of the “PC9-magenta” module in 9,544 cells. Clusters are colored by treatment: orange for DMSO (as a control) and blue for gefitinib (left). Cluster 1 included 1,037 PC9 gefitinib-treated single cells based on their expression profiles of the module “PC9-magenta”. The plot shows cells expressing AURKA alone (green panel), DUSP1 alone (pink panel), and both of them together (yellow panel). The number and ratio of gefitinib-treated cells expressing AURKA and DUSP1 are shown in the inset table. The Fisher’s exact test value is shown in the plot (*p = 0.39).

To characterize the modules, we conducted gene ontology (GO) analysis for each module and selected modules that were associated with particular biological functions. Among them, we first focused on one module, named “lightsteelblue1”, which consisted of 38 genes (Sup. Table S4). Most of these genes were related to cell cycle phase G2/M or cell division (Sup. Table S5). Using a gene belonging to this module, we conducted clustering analysis for the 61 cells (Fig. 7A). By inspecting the constructed heat map, we found that a particular cell, s_062 that had been treated with gefitinib was positioned away (indicated by a red box in Fig. 7A) from the other gefitinib-treated cells. This cell was thus located among the untreated cells in this module. Indeed, the ME value for this particular cell showed a positive value that was similar to that of untreated cells, while all other gefitinib-treated cells showed negative values. We further examined the characteristic features of this cell and found that the gene expression level of the Aurora A kinase (AURKA) gene, which is a member of this module, was significantly elevated in this cell (Fig. 7B). The AURKA gene is a known oncogenic serine/threonine kinase and plays an important role in tumorigenesis and chemoresistance by inhibiting the p53 signaling pathway^45,46^. Interestingly, increased expression of the AURKA gene has been reported to decrease drug sensitivity in several non-small-cell lung cancer cell lines^45^. Gefitinib induces a delay in cell cycle progression, causing G0/G1 arrest and/or G2/M block^47^. Considering these pieces of literal information together, it is likely that the cell s_062 should neutralize the effect of gefitinib via the high expression of AURKA and other genes belonging to this module.

### Possible biological relevance of outlier cells

We further scrutinized the activities of other modules in this particular cell, s_062. We found that the ME value of the cell was extremely high for another module, “magenta” (Sup. Table S6: magenta gene list). This module comprises a group of genes associated with the GO terms of “immune response” (Sup. Table S7). Particularly, we found that cell s_062 highly expressed the Dual specificity phosphatase1 (DUSP1) gene (Fig. 7A, right). The DUSP1 gene encodes a nuclear protein that is highly expressed in heart, lung and liver. By regulating the MAPK pathway^48,49^, this gene promotes angiogenesis, invasion and metastasis in non-small-cell lung cancer cells^50^. Increased expression of DUSP1 has been linked to causing acquired or intrinsic resistance to anti-cancer drugs by regulating apoptotic cell death^51^. Another very recently published study showed that DUSP1 expression levels determine the threshold of TKI efficiency in kinase-driven leukemia^52^. In the s_062 cell, the simultaneous activation of the AURKA-DUSP1 modules may induce the dormancy of the cells, thereby buffering the effect of the drug.

We further characterized the s_062-type cell type as having high expression levels of the AURKA and DUSP1 genes (Fig. 7B). We analyzed the frequency of this cell type within the entire population of PC9 cells using the micro-droplet dataset. We also analyzed how diverse the expression patterns of those cells are within the entire population. For this purpose, we conducted a similar module analysis using the micro-droplet dataset. First, we mapped the 9,544 PC9 cells (5,166 and 4,378 untreated and gefitinib-treated cells, respectively) to the tSNE plane using the expression information of the genes belonging to the module “magenta,” which is the DUSP1 gene module. On this plane, the cells were roughly separated into two clusters, one consisting mostly of the untreated (cluster 2) cells and one consisting mostly of gefitinib-treated (cluster 1) cells (Sup. Fig. S7). Similar to the observations from the analysis using the micro-chamber dataset, some of the gefitinib-treated cells were identified within the cluster of untreated cells (Fig. 7C, left). To examine whether those gefitinib-treated cells had features similar to the cell s_062, we examined whether those cells expressed both the AURKA and DUSP1 genes. We found that high expression levels of the AURKA genes emerged among the gefitinib-treated cells at a similar region in the plane (Fig. 7C, right, indicated by the yellow “merged” panel). Namely, 92 cells (8.8% of the gefitinib-treated cells in cluster 2) showed high expression levels of both the DUSP1 and AURKA genes (Fig. 7C, inset table). Further statistical evaluation showed that the overlapping pattern of the expression of these genes revealed independent aberrant upregulation, although the overall expression patterns of double-positive and single-positive cells were frequently similar (Fig. 7C inset table, Fisher’s exact test, p = 0.39).

### Module activities in other cell lines

**Figure 8:**
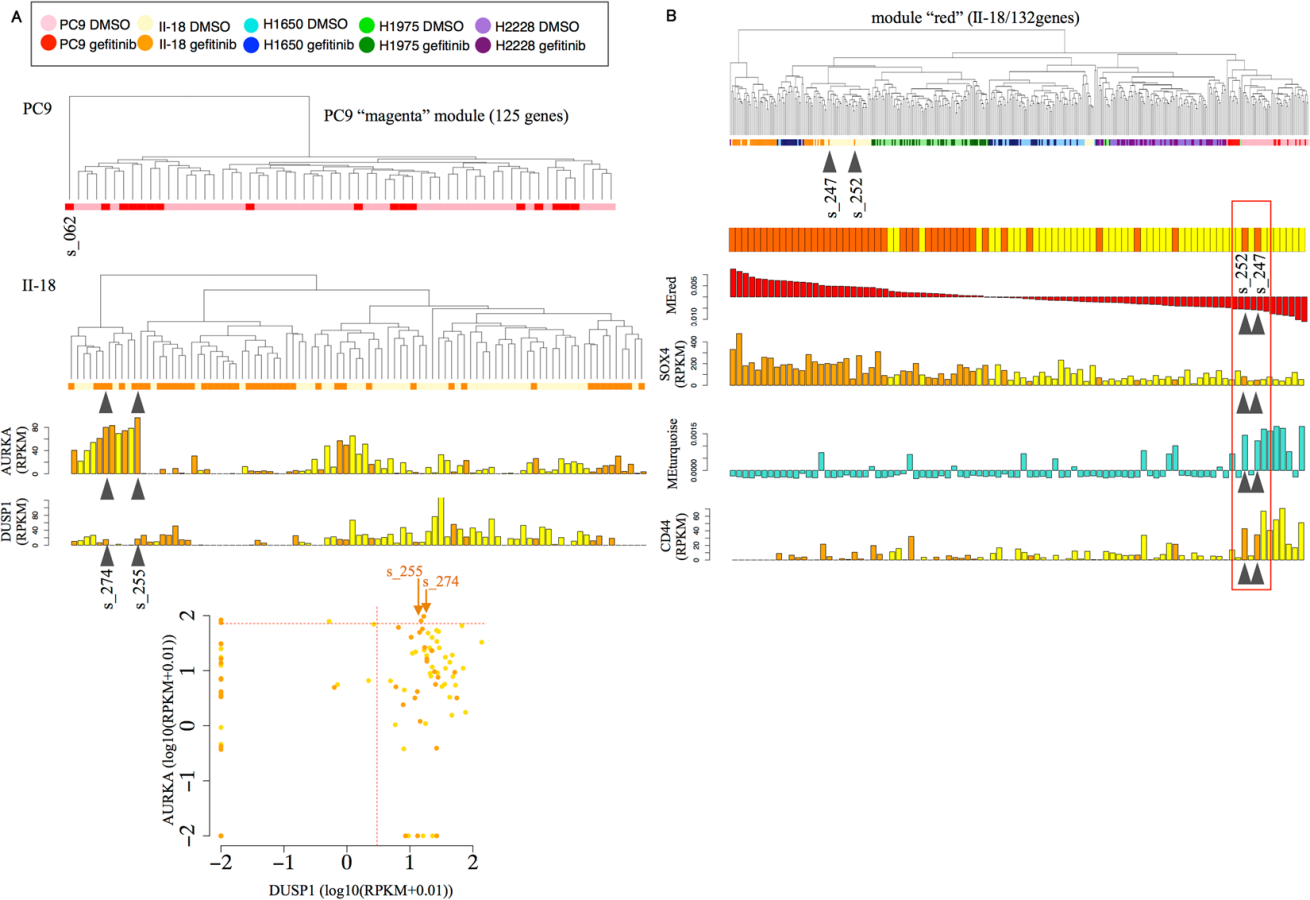
Module analysis in another cell line. (**A**) Hierarchical clustering analyses were conducted using the genes included in the “magenta” module in PC9 (top) and II-18 cells (second). The expression levels (RPKM) of AURKA and DUSP1 are also shown in the bar plot (middle). The scatterplot shows the relationship between the expression levels of two genes, DUSP1 (*x*-axis) and AURKA (*y*-axis) (bottom). (**B**) Clustering of II-18 cells by the module “II-18-red.” Hierarchical clustering analyses were conducted using the genes included in the II-18 module “red” (top). The treatment of individual cells, their MEred value, their expression level of SOX4, their MEturquoise value and their CD44 expression level are shown. Two cells (s_252 and s_247) show low expression of SOX4 and high expression of CD44.

We examined whether the high activities of the DUSP1-AURKA gene modules were unique to gefitinib-treated PC9 cells. Using the micro-chamber datasets for the other cell lines, we conducted a similar module analysis. First, we performed clustering analysis using entire genes and found that the cells were separated depending on their originating cell types (Sup. Fig. S8A). Of note, the cells were not separated between untreated and gefitinib-treated cells in H1975 and H2228, which gefitinib should not affect (Sup. Fig. S8B). Distinct patterns were observed for the PC9 and II-18 cells, among which the untreated and gefitinib-treated cells were distinct. H1650 cell clustering was marginal, perhaps reflecting their partial response to gefitinib. When we clustered each of the cell lines by the “magenta” module (the DUSP1 gene module) in PC9 cells, we did not identify clear outlier cells in the gefitinib-treated II-18 cells. Several cells had high expression levels of AURKA and DUSP1 genes (s_255 and s_274 cells; indicated by arrows; Fig. 8A). However, their expression levels were less significant than those of the outlier PC9 cell, s_062 (top panel; Fig. 8A).

Instead, when we conducted a similar analysis for II-18 cells, we identified a distinct module, “red.” This module consisted of 132 genes (Sup. Table S8) with the SOX4 gene as a core. Clustering using this module identified two outlier cells (s_252 and s_247 cells) (Fig. 8B) that exhibited high levels of ME-turquoise and CD44 expression. Analysis using the micro-droplet dataset revealed that such cells represented 0.06% of the entire population (Sup. Fig. S9). SOX4 has been reported to act as a tumor suppressor gene, depending on the context, by promoting cell cycle arrest and apoptosis^53–55^. CD44 is known as a cancer stem or cancer progenitor marker in several tumors^56,57^. Another report indicates that cells expressing CD44 show stem-cell-like properties^58^. Aberrant activation of this module was not observed in PC9. Cancer cells may utilize some common modules for survival but may more frequently use unique modules, depending on their original transcriptomic status.

### Possible biological relevance of outlier cells

**Figure 9:**
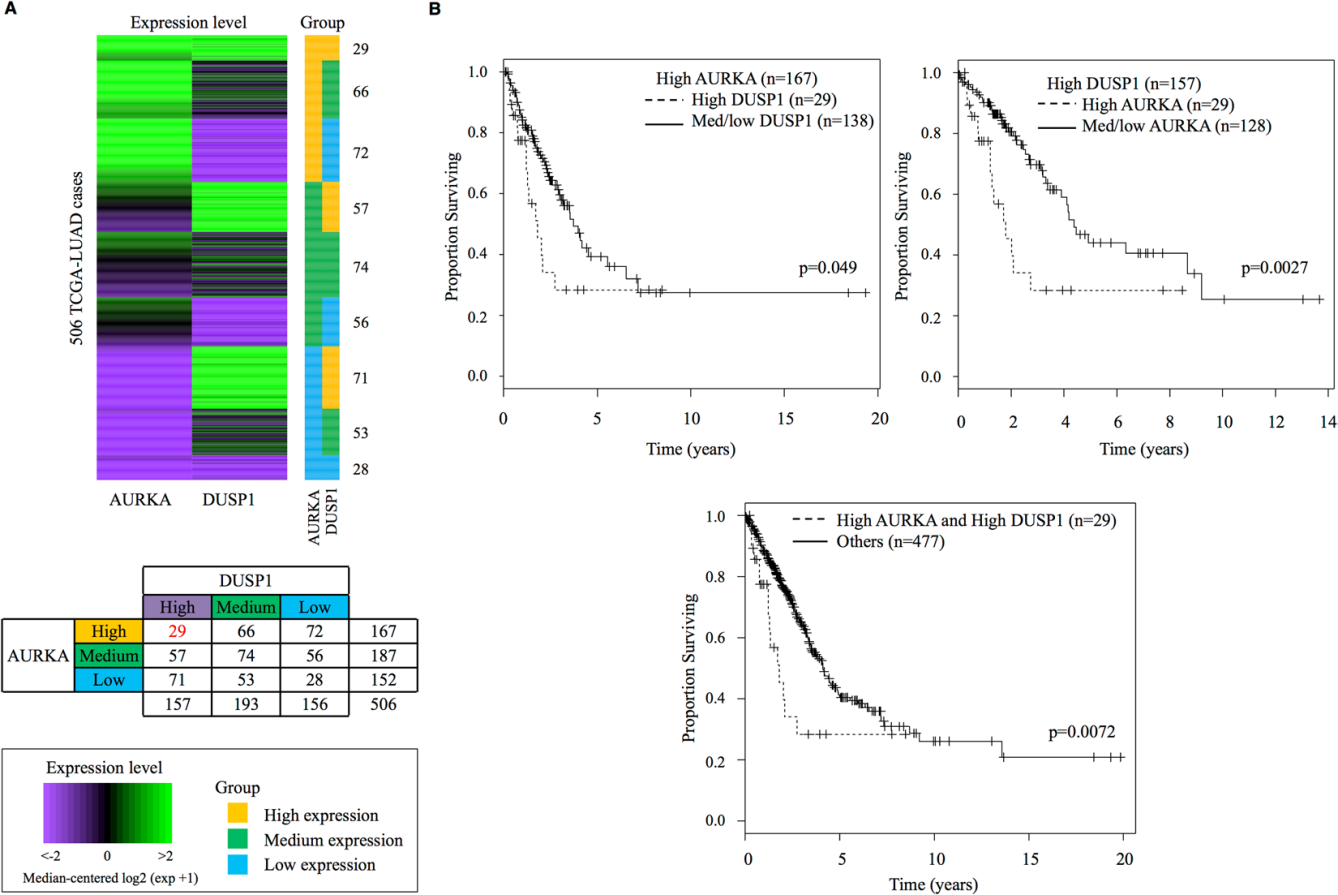
Biological relevance based on the TCGA-LUAD dataset. (**A**) Heatmap of 506 TCGA patients, showing their expression levels of AURKA and DUSP1. The patients were divided into nine groups based on the expression profiles. In the right margin, the groups of patients are shown (top). The number of patients in each group is shown in the inset table (middle). The color bar in the box shows the gene expression levels and the groups (bottom). (**B**) The Kaplan-Meier curve shows that patients with high expression levels of both AURKA and DUSP1 are associated with a poor prognosis. Statistical significance (p-value) of differences between the two groups is shown in the plot.

Using data from clinical samples, we investigated the potential phenotypic significance of the outlier cells with high expression levels of DUSP1 and AURKA genes. For this purpose, we used the TCGA dataset^9^, which provides transcriptome information as well as clinical information for 506 lung adenocarcinoma patients. We divided the patients into two groups based on AURKA and DUSP1 expression levels (Fig. 9A, inset table). Again, there seemed to be no or little direct correlation between the expression levels of these genes in the clinical samples. Twenty-nine cases showed high expression levels of both genes. We compared the overall survival times of the patients depending their expression levels of AURKA and DUSP1 genes. We observed that the patients with high expression levels of both AURKA and DUSP1 genes showed a poor prognosis compared with cases with normal or low expression levels of either gene (Fig. 9B). Activation of AURKA and DUSP1 genes may have a favorable effect on the survival of lung cancer cells. Outlier cells with modules highly related to AURKA and DUSP1 would have survival advantages, particularly under severe conditions, and may contribute to the development of small populations of more malignant cells for which anti-cancer drugs are less effective.

## Conclusions

In this study, we first evaluated the representative two analytical platforms, the micro-chamber and the micro-droplet methods, which are used for scRNA-seq. We found that the datasets obtained from those different platforms have the similar nature, although the respective platforms have their unique advantages and disadvantages. To make the most use of the advantages of these two platforms, we attempted to combine the datasets generated from two different platforms in the later sections. In the first part of the paper, we generated datasets from the micro-chamber and the micro-droplet platforms. In either of the platforms, the datasets consisted of the gene expression information of each individual single cell. Such a separation was possible even though the two platforms identify the single cell based on the different methods. Namely, for the micro-chamber platform, we collected the individual cells by separating the cells into different chambers. With the micro-droplet platform, we separated the cells by confining the cells into micro-droplets, where the mRNAs of each cell were labeled with distinct barcodes in individual droplets. As a result, even though the number of the analyzed cells and the sequencing depth per cell differed between the two platforms, we could compare the expression information between the data at the individual cell level. Detailed technical evaluations and comparisons of these platforms revealed that both methods were highly reproducible and concordant. However, the difference in sequence depths, which depends on the number of cells subjected to the analysis with a given sequencing cost, caused distinct features of the datasets. Indeed, both methods had inherent advantages and disadvantages. Namely, the micro-chamber system enabled us to examine the detailed character of each cell in terms of its gene expression information. However, the feasible number of subjected cells is too small to detect a rare population of cells and estimate the frequency and variance of those cells if they are detected. Conversely, the micro-droplet platform examined a much larger number of cells. We considered that the micro-droplet platform to be still useful, despite the expression information from a given single cell being relatively poor. This is the only currently available platform that can analyze >5,000 cells at the same time. Without this platform, it would be essentially impossible to characterize a population of cells regarding the gene expression information of each individual cell. Therefore, that the micro-chamber method should be complementarily used with the micro-droplet method. Namely, the former has an advantage in the sequencing depth and rich expression information for each single cell, while the latter has an advantage in the population analysis of the cells. However, the limited sequencing depth for each cell makes interpretation of the gene expression data on its own difficult.

In the second part of this paper, we demonstrated to address these limitations by integrating the data from the two platforms. First, we provided a statistical inference originating from the micro-chamber dataset that could predict the missing values in the micro-droplet dataset. Second, we identified a minor population of cells using a transcriptional module-based approach with the micro-chamber dataset. Further analyses using the micro-droplet dataset revealed the frequency and divergence of such cells in the entire population. In particular, we identified two modules with the AURKA gene and the DUSP1 gene as their cores. Interestingly, simultaneous activation of those genes was associated with the poorest prognosis in clinical samples. We believe that single-cell analysis would provide indispensable information for further analysis of the molecular basis underlying the emergence of such cancer cells.

Further detailed evaluations are clearly needed to validate the clinical relevance of the observed heterogeneity of cancer cells. Diverse *in vivo* microenvironments should further impose complicated factors on cellular gene expression. Several methods to monitor single-cell transcriptomes *in vivo* are being developed. However, the resolution and precision of the data are still limited. Taking various advantages of the cell lines, we believe that this work should provide a first step towards a thorough understanding of the diverse nature of cancer.

## Materials and Methods

### Cell culture

PC9 and II-18 cells were acquired from the RIKEN Bio Resource Center (catalog number RCB4455 and RCB2093), and H1650, H1975 and H2228 were acquired from the American Type Culture Collection (catalog numbers CRL5883, CRL5908 and CRL5953). The cells were grown in RPMI-1640 medium (Wako, 189–02145) with 10% fetal bovine serum (FBS), MEM Non-Essential Amino Acid Solution (catalog number M7145, Sigma-Aldrich, St. Louis, MO) and penicillin and streptomycin in an incubator maintained at 37 °C with 5% CO_2_. For gefitinib (CAS 184475-35-2, Santa Cruz Biotechnology) treatment, the drug was added to the culture medium at a final concentration of 1 μM. Twenty-four hours after the drug treatment, the cells were harvested. For the untreated control, DMSO was added to the culture medium in place of gefitinib. For each experiment, 10^6^ cells were harvested and separated using bead-seq and a Chromium Single Cell 3’ (10× Genomics, version 1).

### Single-cell RNA-seq with the micro-chamber system

We prepared libraries according to Matsunaga *et al*.^31^ and utilized the HiSeq. 2500 platform (Illumina) with 50-base single-end reads. For the PC9 replicate samples, we performed 35-base single-end reads. To remove ribosomal RNA, the generated RNA-seq tags were mapped to rmRNA, and unmapped reads were removed. Trimmed reads were aligned to the human reference genome (UCSC hg19) by TopHat/Bowtie. Using our Perl script, RNA-seq tag counts were calculated as reads per kilobase RNA per million mapped tags (RPKM)^59^.

### Single cell RNA-seq with the micro-droplet system

Using Chromium Single Cell 3′, libraries were prepared according to the manufacturer’s instructions. We used a HiSeq. 2500 Rapid run platform to generate 50-base paired-end reads. RNA-seq tags from the Chromium experiments were aligned using Cell Ranger software. Using our Perl script, sequences with low quality and PCR duplicates were removed. Trimmed reads were sorted based on their cell barcode, and only cell barcodes with >5 k tags were selected. Using our Perl script, RNA-seq tag counts were calculated as parts per million mapped tags (ppm).

### Correlation analysis between two platforms

When the values of the results from the two different platforms, which have distinct numbers of cells and sequencing depths, were compared, the statistical significance of the difference was evaluated by the indicated methods. For the correlation analysis at the cell to cell level, we selected the individual cells having the largest the second largest and the third largest number of their sequence tags and designates them as “top1”, “top 2” and “top3” cells, respectively, for each of the platforms.

### Cell cycle analysis of PC9 cells

As shown in Fig. 5A at the top left, we used 44 PC9 DMSO-treated cells and 20 cell cycle-regulated genes (four genes per phase) to refine the cell state; CCNE1, E2F1, CDC6 and PCNA were used for G1/S phase, RFC4, DHFR, RRM2, and RAD51 for S phase, CDC2, TOP2A, CCNF and CCNA2 for G2 phase, STK15, BUB1, CCNB1 and PLK1 for G2/M phase, and PTTG1, RAD21, VFGFC and CDKN3 for M/G1 phase. These gene sets were obtained from Whitefield *et al*.^60^. The expression levels (RPKM) of each gene in the gene set of each single cell were calculated and scaled. To order the cells, we compared the average scores of five phases.

For the micro-droplet datasets, we first attempted to draw a heatmap using same method as for the micro-chamber dataset. However, we cannot draw the heatmap as in Fig. 5A top due to the absence of values in the micro-droplet datasets. To overcome those problems, we analyzed the cell cycle of each cell based on the method previously reported by Macosko *et al*.^33^. As shown at the bottom left of Fig. 5A, we used 5,166 PC9 DMSO-treated cells and 603 genes from Macosko *et al*. From those genes, we excluded genes with a low correlation to the cell state (r < 0.2). Twenty-one genes correlated with the G1/S phase, 14 genes with the S phase, 39 genes with the G2/M phase, 51 genes with the M phase, and 19 genes with the M/G1 phase remained (a total of 144 genes, Sup. Table 2). We calculated the expression levels of these genes and averaged the normalized (log2(PPM+1)) values in each phase. We scaled these scores and obtained a phase-specific score for 5,166 cells. Next, we compared the pattern of the phase-specific scores to nine potential patterns to determine the cell phases and ordered the cells according to their phases. Of the 5,166 cells, 2,812 cells were grouped into five phases. In contrast, the other 2,354 cells were estimated to be intermediate between G1/S and S, G2/M and M, and M/G1 and M phases. With the ordered datasets, we ran the R package “gplots” and used the “heatmap2” routine included in this package^61^.

At the top right of Fig. 5A, we used the same datasets as in the heatmap. At the bottom left of Fig. 5A, we used the 2,812 cells that had been grouped into five phases. We did not use the cells estimated to be intermediate between phases. To generate a two-dimensional projection, we reduced the dimensionality of those two datasets by principal component analysis (PCA)^62^. We represented individual cells by running R package “ggplot2” to draw figures^63^.

### MAPK Analysis of PC9 cells

To determine the expression levels of genes included in the MAPK/ERK pathway in Fig. 5B, we mapped the tag counts of each gene in the illustration^43^. We used the top1 cell, the cell with the largest number of mapped reads per cell, from each platform to color the figures^64^.

### Estimation of missing values

To estimate missing values, we combined gene expression data from two systems. We used 232 DMSO-treated cells and 210 gefitinib-treated cells from the present study. Genes with an average RPKM >10 across different cells were selected from the micro-chamber data sets. We also used the micro-droplet system datasets as predictors and to construct predictive models. The base-10 logarithms of all the expression levels were processed, and a pseudo value of 0.01 was used for values that were missing before the logarithms of the values were taken. There were 4,901 and 4,845 genes in the micro-chamber system with RPKM >10 for the DMSO- and gefitinib-treated cells, respectively. The expression levels of the genes in the micro-chamber system were encoded as explanatory variables, and the other genes that were not consistently among the explanatory variables were encoded as response variables. LASSO regression was then performed^65^. The response functions of LASSO were subsequently employed with the micro-droplet system datasets to predict gene expression levels.

To validate the estimation, we used the gene expression levels of the micro-droplet system dataset that were not missing and compared them with the values that had been estimated according to the computational method (Fig. 6A and D). The global correlation coefficients were determined by calculating Pearson’s *r* between the experimental values and predicted values of all the cells.

All the R programs were executed using R version 3.3.1, and the R package “glmnet” was employed to perform the Lasso regression. The parameter lambda in the Lasso regression was set to the 10th value of the lambda list in “glmnet” R package, and other parameters were set to their default values^66^.

### Module-based single-cell analysis

We ran R package “WGCNA” and estimated co-expression network modules. First, we used 66 cells (DMSO-treated and gefitinib-treated PC9 cells)^44^. We clustered the samples and detected and removed five outlier cells with low expression levels (<5 RPKM) for more than 5000 genes. We removed genes that were not expressed much more than 5 RPKM in at least one cell. Based on the scRNA-seq data from 61 PC9 cells, we identified 71 modules and listed the genes included in those modules and the ME value of each cell. To evaluate the characteristics of these modules, we also conducted an eigengene network analysis and gene ontology (GO) enrichment analysis, which are included in the WGCNA package. We repeated the same process for the other four cell lines: II-18, H1650, H1975, and H2228. Figures were generated based on the identified modules (Sup. Table S9).

To create Fig. 7A, we used 61 PC9 cells (44 DMSO-treated and 17 gefitinib-treated cells) and the expression levels of genes included in the module “lightsteelblue1”. First, we rearranged the cells in the MElightsteelblue1 value order and represented the treatment (DMSO or gefitinib) and MElightsteelblue1 value for each cell with a bar plot. We then transformed the expression level of the gene in the module “lightsteelblue1” to a log2(RPKM+0.01) value and drew a heatmap. We used heatmap.2, which is included in the R package “ggplots.” In the right margin, we show the expression levels of four genes, the top3 module genes and AURKA, and the MEmagenta value for each cell with a bar plot.

To create Fig. 7C, we used the expression levels of the genes included in the module “magenta.” We projected 9,544 cells based on their PC scores onto a two-dimensional map using t-Distributed Stochastic Neighbor Embedding (t-SNE)^67^. Cells were clustered into two clusters based on the k-means score and colored by treatment, orange for DMSO and blue for gefitinib.

To create Fig. 8, we gathered data from 429 cells (Sup. Table 5) and applied a hierarchal clustering based on the genes included in the modules “II-18-red” (top) and “magenta (PC9 module)” (bottom).

### Survival analysis

To analyze the TCGA dataset, we downloaded the RNA-seq v2 data and clinical information for the TCGA lung adenocarcinoma (TCGA-LUAD) dataset from the NCI Genomic Data Commons using TCGA-Assembler v2.0.1 (the data downloaded on 2017/03/09)^68^. We obtained 506 cases with both RNA-seq and clinical data. As the RNA-seq dataset, we downloaded the dataset by TCGA-Assembler with the following options; assayPlatform = “gene.normalized_RNAseq” and cancerType = “LUAD.” We transformed the expression levels to their log2(expression + 1) values. In the present study, we denoted expression “high” when its level was > average + 0.5 s.d. and “low” when its level was < average −0.5 s.d. We download the clinical dataset by TCGA-Assembler with the option; cancerType = “LUAD.” The data for overall survival for each case were extracted from clinical patient and follow-up files. Kaplan-Meier analysis with the log-rank test was conducted using the survival package in R^69^.

### Availability of data and material

The sequence data from this study have been submitted to DNA Data Bank of Japan under accession number DRA005922- DRA005929.

## Electronic supplementary material

supplemental information
