# Supplementary material for: Combinatory use of distinct single-cell RNA-seq analytical platforms reveals the heterogeneous transcriptome response
Source: Sci Rep. 2018 Feb 22;8:3482. doi: 10.1038/s41598-018-21161-y (PMC5823859; doi:10.1038/s41598-018-21161-y)
Supplement: Supplementary file 1 — supplemental information [file 41598_2018_21161_MOESM1_ESM.pdf]

Combinatory use of distinct single-cell RNA-seq analytical platforms reveals the heterogeneous transcriptome response

Yukie Kashima, Ayako Suzuki, Ying Liu, Masahito Hosokawa, Hiroko Matsunaga, Masataka Shirai, Kohji Arikawa, Sumio Sugano, Takashi Kohno, Haruko Takeyama, Katsuya Tsuchihara and Yutaka Suzuki

SUPPLEMENTAL MATERIALS

SUPPLEMENTAL FIGURES S1-S9 pp2-pp20
SUPPLEMENTAL TABLES S1-S9 pp21-pp29

A

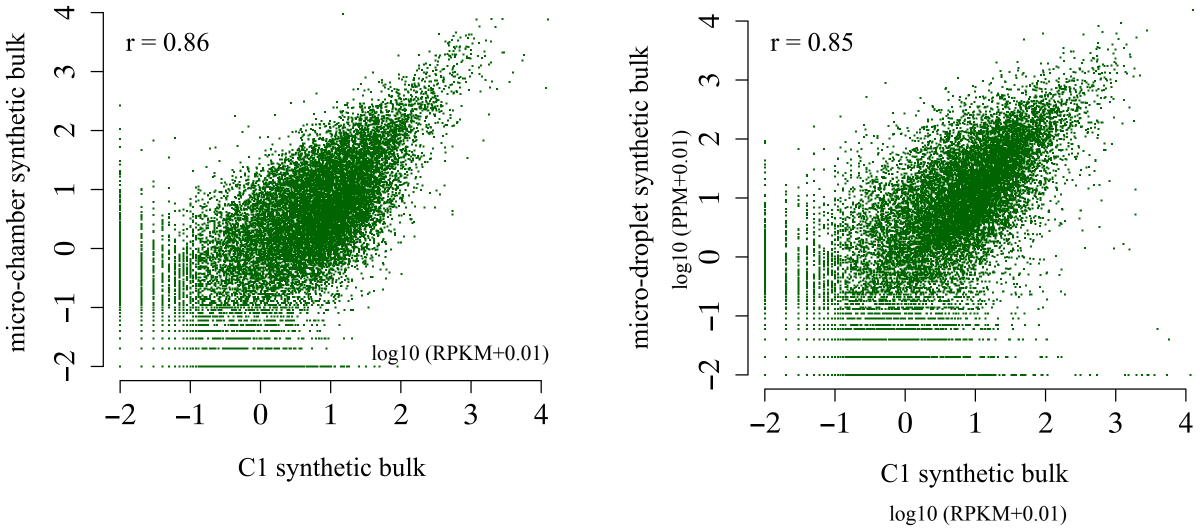

B

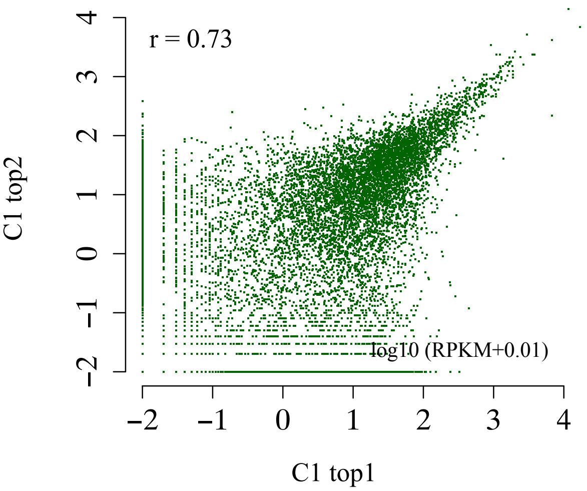

C

Cell line	sample	Raw Read/cell	used reads/cell	Number of Cells
PC9	average	21,669,191	14,455,179	47
	top1	30,704,526	20,239,586	1
	top2	30,607,350	21,852,050	1

Supplemental Figure S1: Generation of the RNA-Seq dataset using the micro-chamber system, micro-droplet system and C1 Fluidigm

(A) Comparison between the synthetic bulk dataset for the two platforms, C1 Fluidigm (*x*-axis) and micro-chamber system (*y*-axis) (left), and C1 Fluidigm (*x*-axis) and the micro-droplet system (*y*-axis) (right). The Pearson's correlation between the two experiments is shown in the plot. (B) Comparison between two single-cell datasets by C1 Fluidigm. The Pearson's correlation between the two experiments is shown in the plot. (C) Statistics for the C1 Fluidigm system data used in the present study.

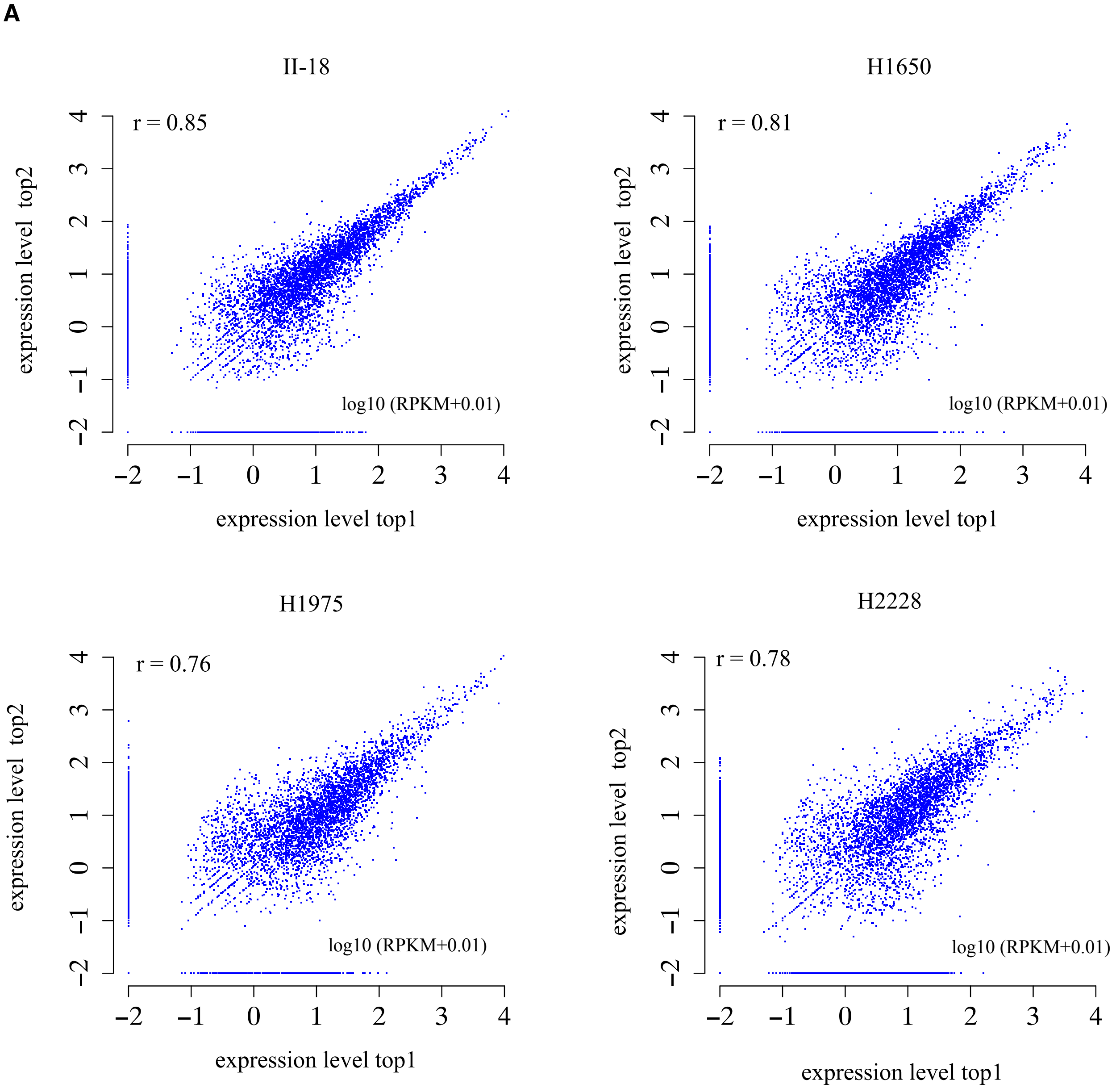

Supplemental Figure S2: Generation of RNA-seq dataset using two platforms
(A) Comparison of the expression level between top1 (*x*-axis) and top2 (*y*-axis) using the micro-chamber system in four cell lines: II-18, H1650, H1975 and H2228. The Pearson's correlation between two experiments is shown in the plot.

B

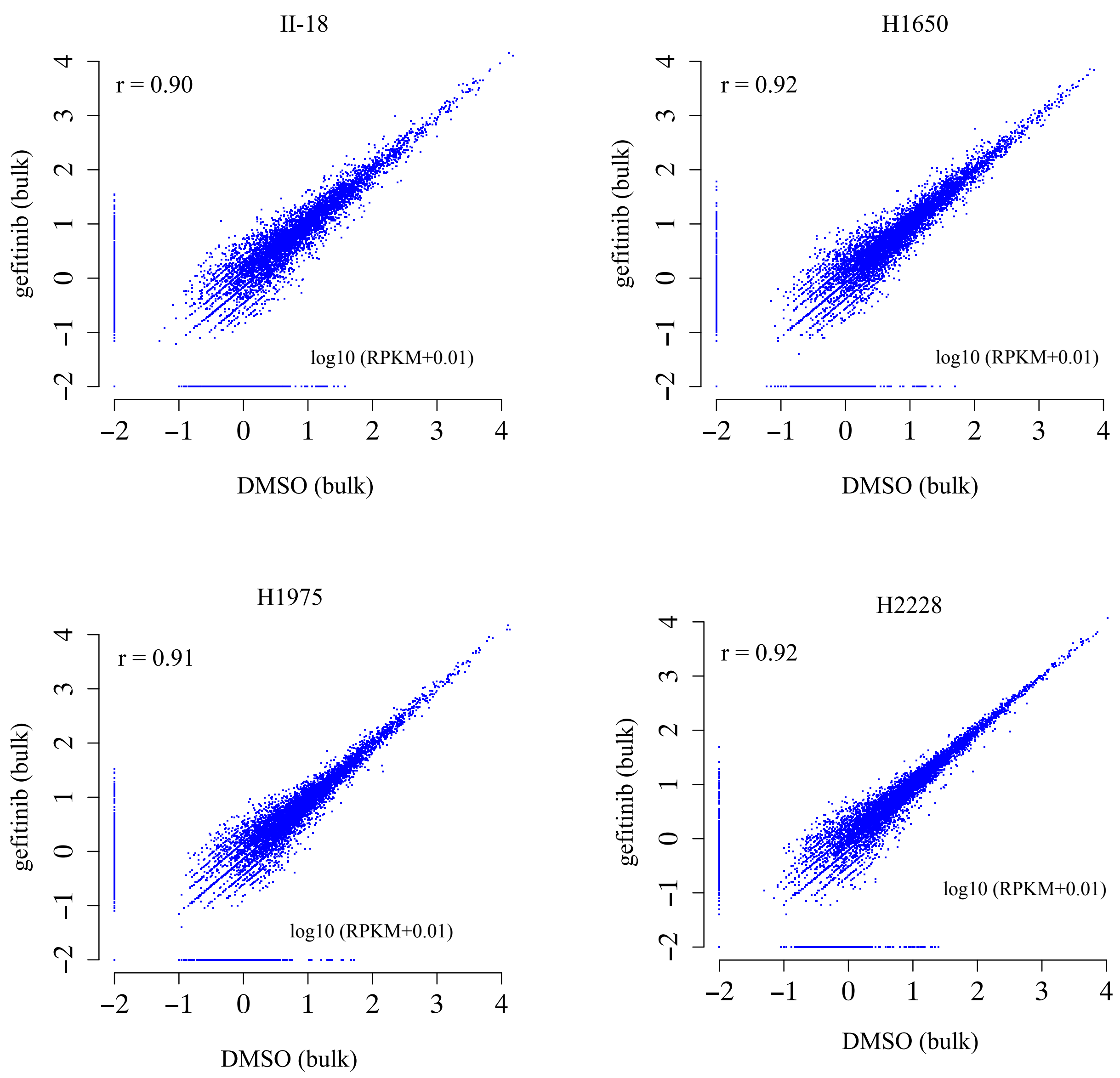

Supplemental Figure S2: Generation of RNA-seq dataset using two platforms
(B) Comparison of the bulk expression level between samples treated with DMSO (*x*-axis) and gefitinib (*y*-axis) using the micro-chamber system in five cell lines: II-18, H1650, H1975 and H2228. The Pearson's correlation between two experiments is shown in the plot.

C

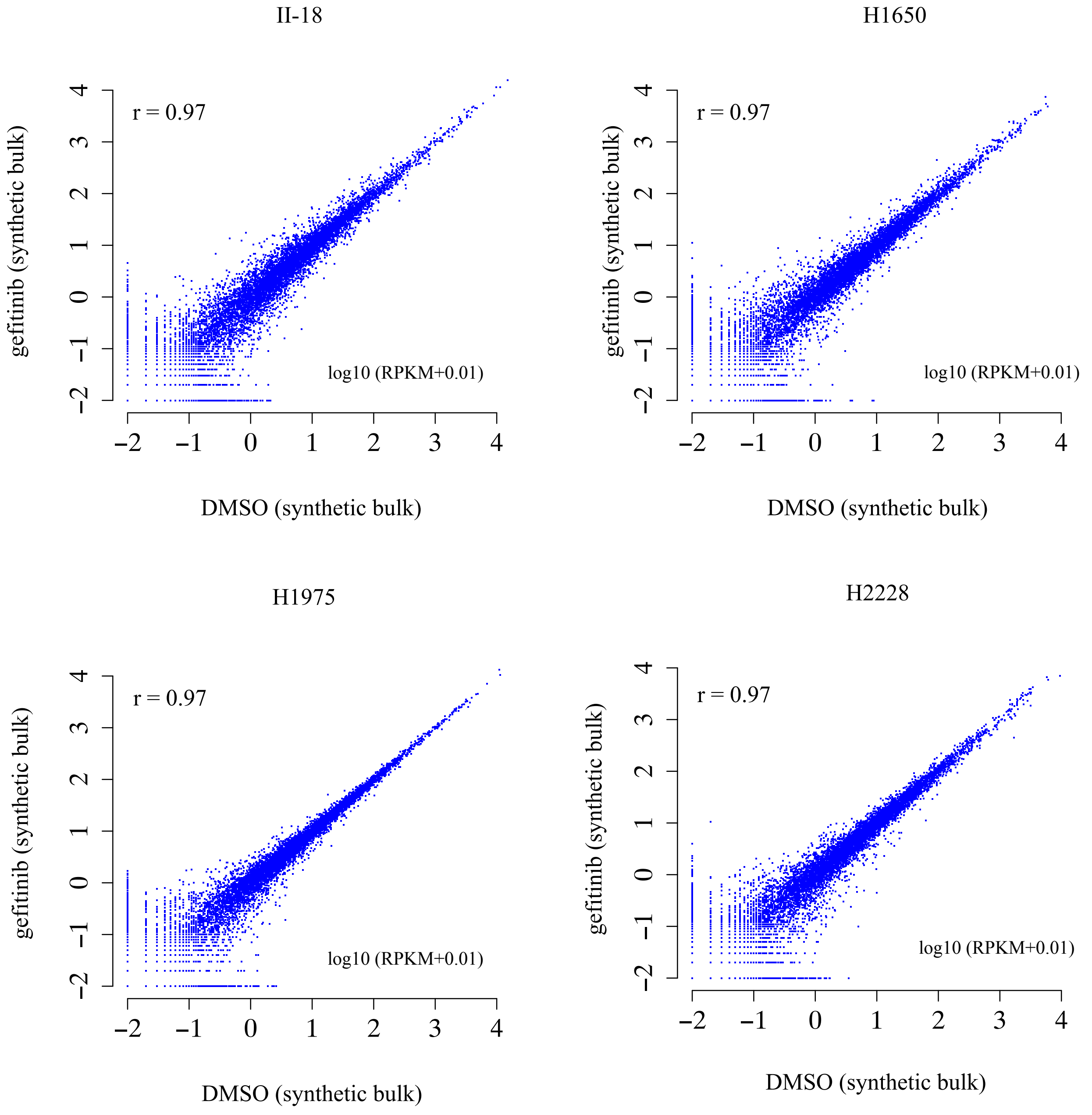

Supplemental Figure S2: Generation of RNA-seq dataset using two platforms
(C) Comparison of the synthetic bulk expression level between samples treated with DMSO (x-axis) and gefitinib (*y*-axis) using the micro-chamber system in five cell lines: II-18, H1650, H1975 and H2228. The Pearson's correlation between two experiments is shown in the plot.

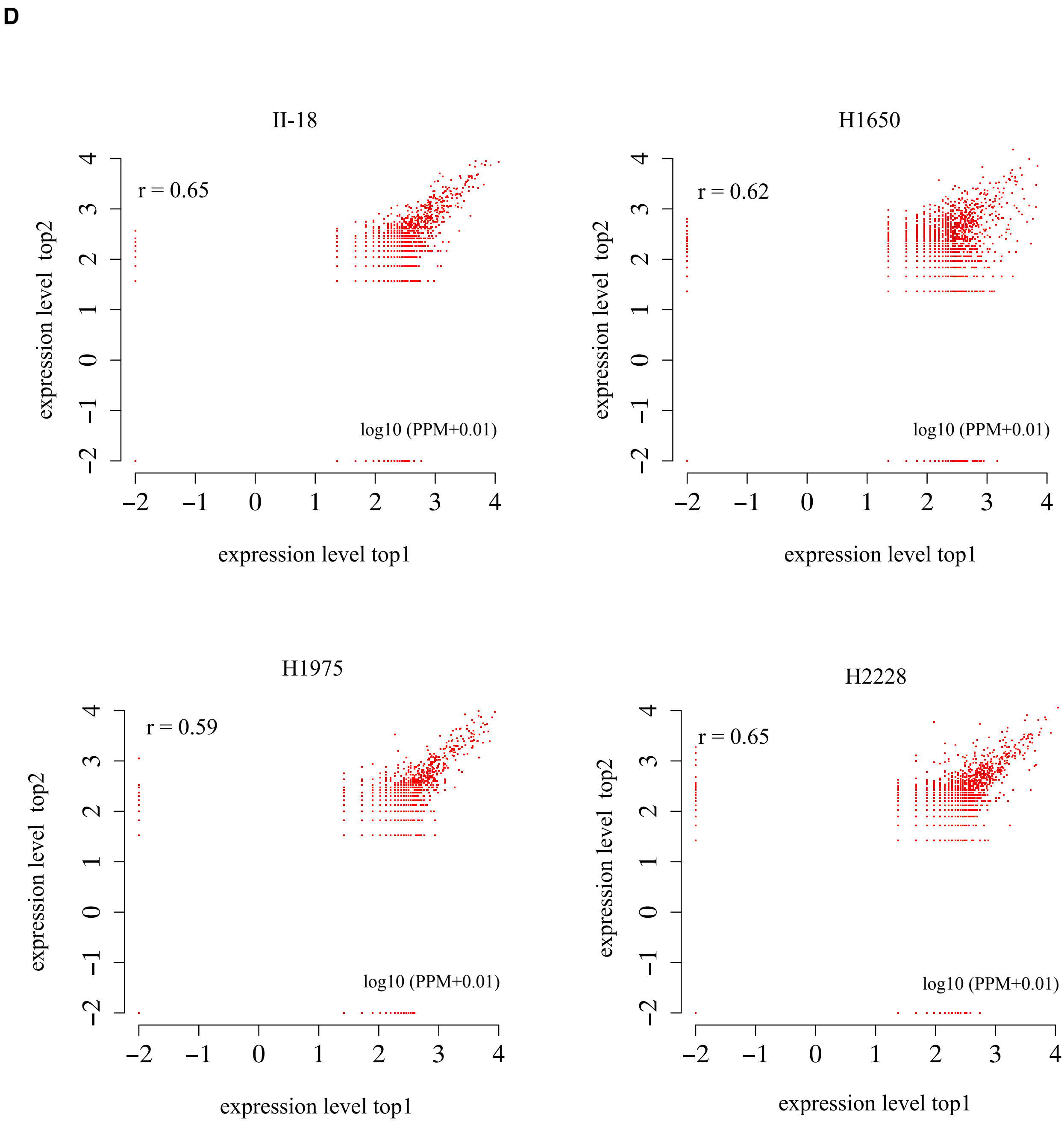

Supplemental Figure S2: Generation of RNA-seq dataset using two platforms
(D) Comparison of the expression level between top1 (*x*-axis) and top2 (*y*-axis) using the micro-droplet system in five cell lines: II-18, H1650, H1975 and H2228. The Pearson's correlation between two experiments is shown in the plot.

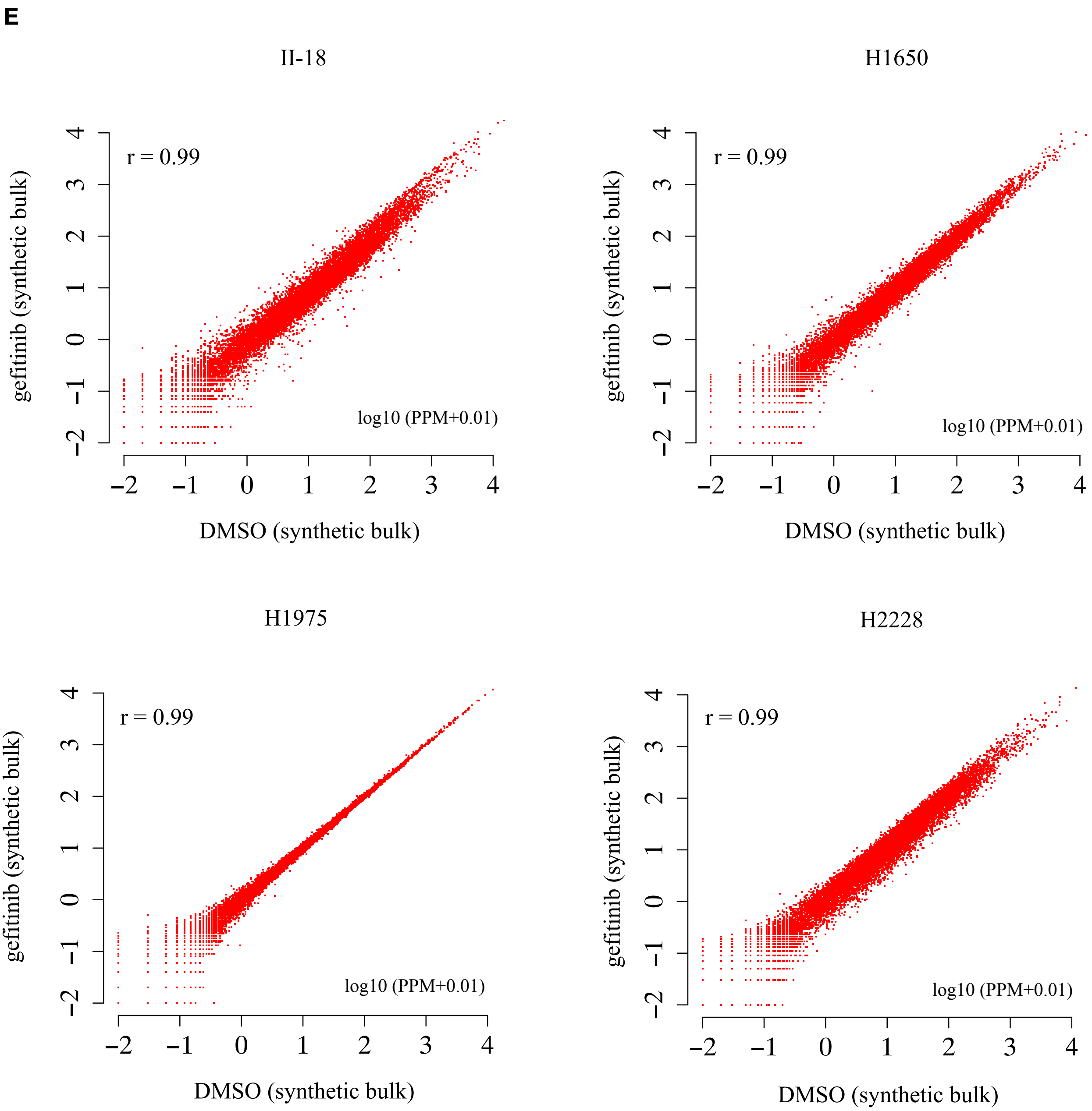

Supplemental Figure S2: Generation of RNA-seq dataset using two platforms

(E) Comparison of the synthetic bulk expression level between samples treated with DMSO (*x*-axis) and gefitinib (*y*-axis) using the micro-droplet system in four cell lines: II-18, H1650, H1975 and H2228. The Pearson's correlation between two experiments is shown in the plot.

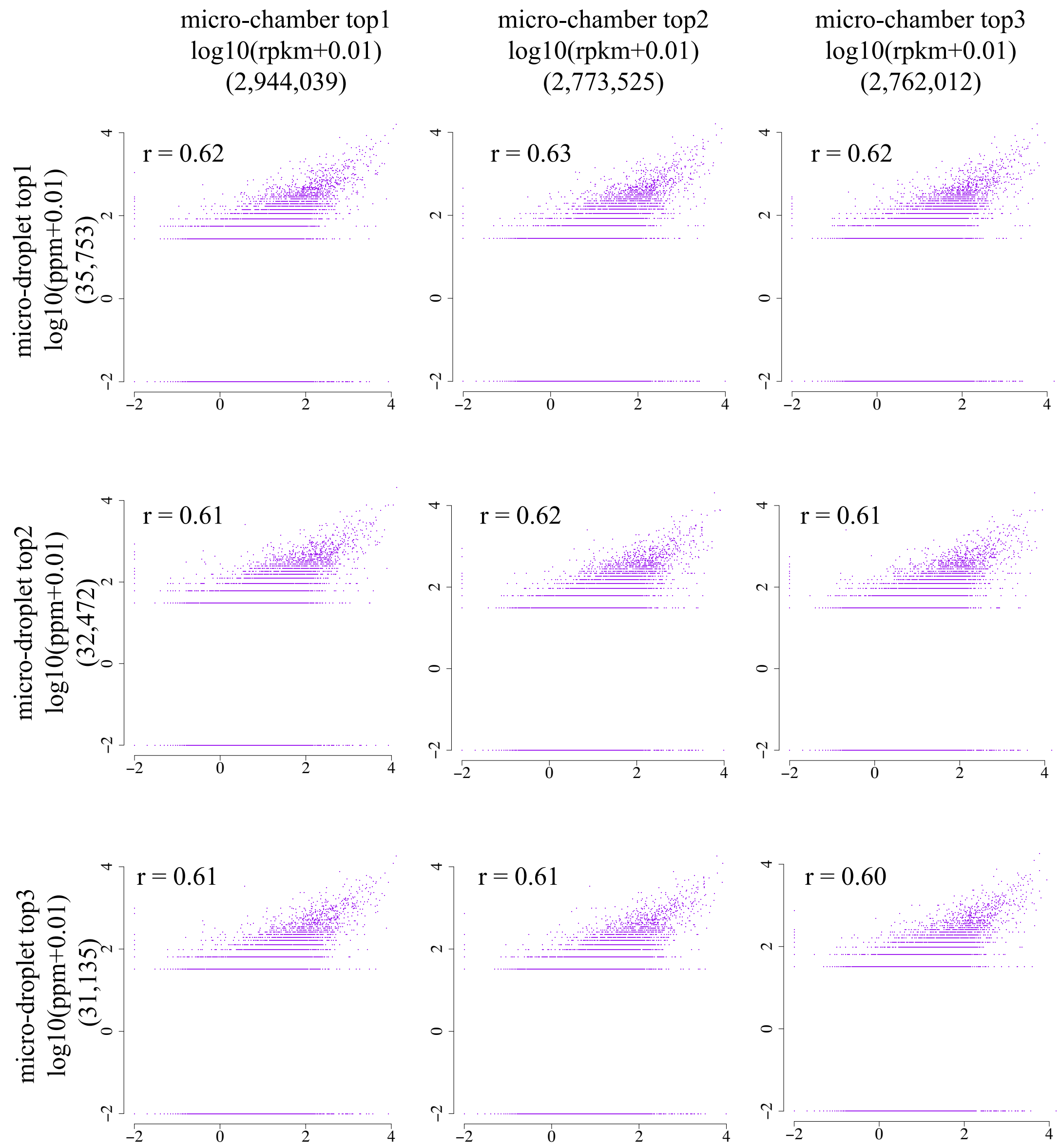

Supplemental Figure S3: Comparison between two single-cell in different platforms

Comparison between single-cell expression of the micro-chamber system (*x*-axis) and the micro-droplet system (*y*-axis) shown in the plot. We selected eight cells with the first, second, third and fourth largest number of mapped scRNA-seq tags (the top1 to top3 cells) from the respective platforms. We calculated and compared expression levels of all genes. The Pearson's correlation between the two samples is shown in the plot.

A

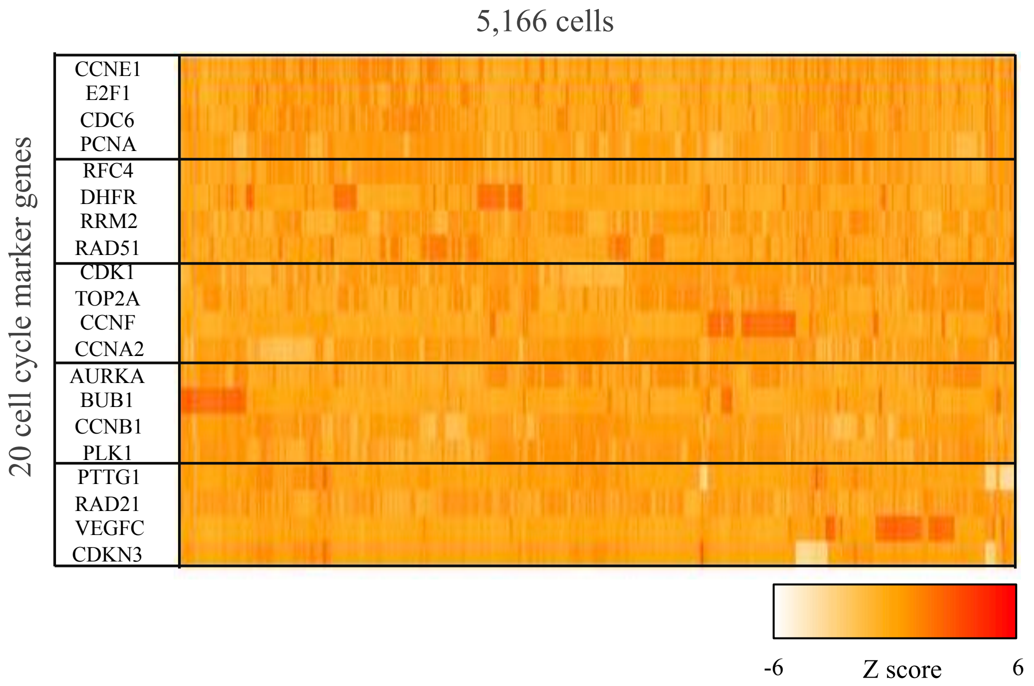

B

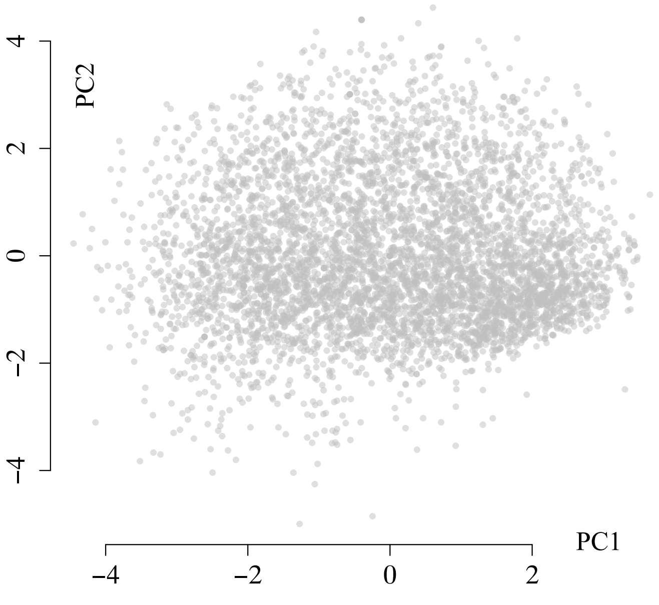

Supplemental Figure S4: Cell cycle status of the micro-droplet dataset estimated by 20 genes
Cell cycle analysis of PC9 cells from the micro-droplet system datasets based on the expression levels of 20 genes. The heatmap (A) and PCA analysis (B) are shown.

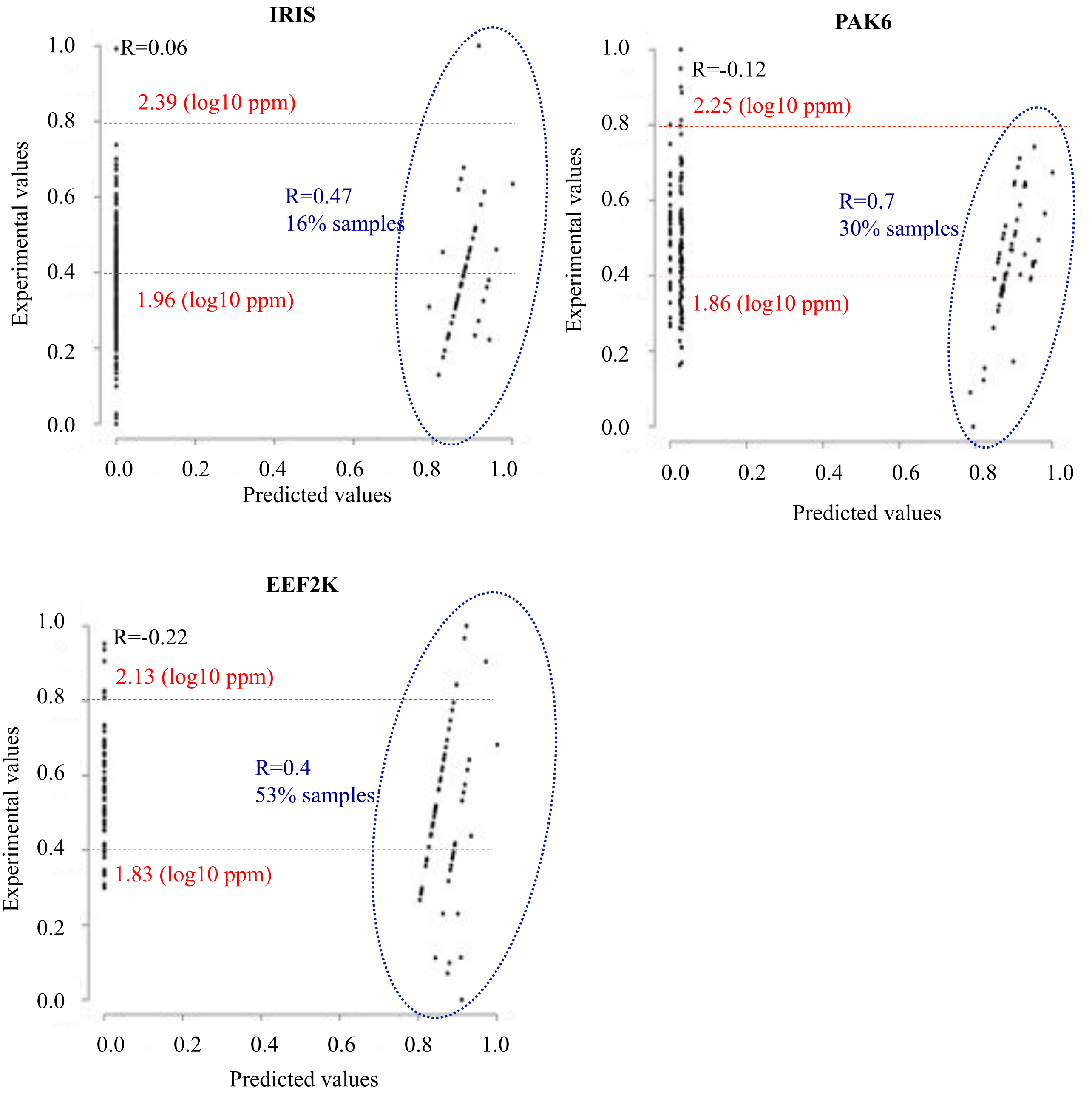

Supplemental Figure S5: Estimation of missing value for genes in the MAPK/ERK pathway
Comparison between normalized predicted values (*x*-axis) and normalized experimental values (*y*-axis) for three genes. We used 0-1 normalization for different scales. The Pearson's correlations between two values of all cells (black) and selected cells (blue) are shown in the plot. Experimental expression levels (log10 PPM) corresponding to 0.8 and 0.4 are also shown in the plot (red).

A

WGCNA network : PC9

micro-chamber dataset 22913 genes/ 66 cells
--> remove outlier cells
(s_046, s_048, s_053, s_054, s_057)
--> remove low expression genes
(5rpkm> at least 1 cell)
--> 13,619 genes / 61 cells
(DMSO 44 cells, gefitinib17 cells)

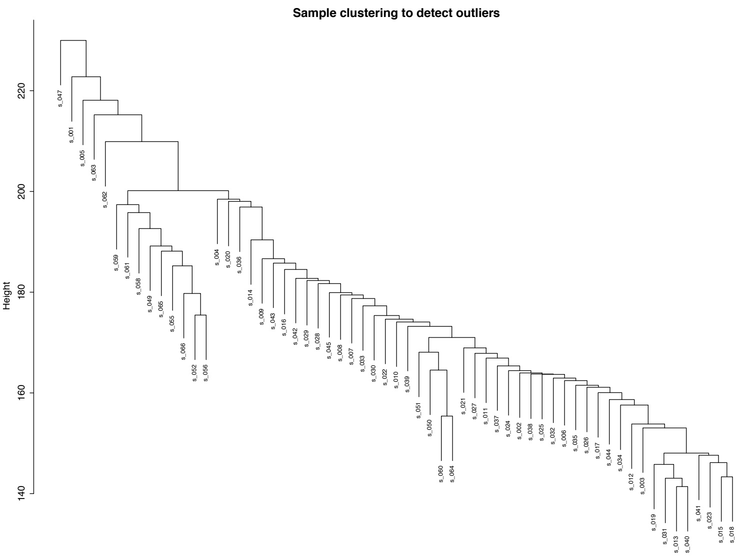

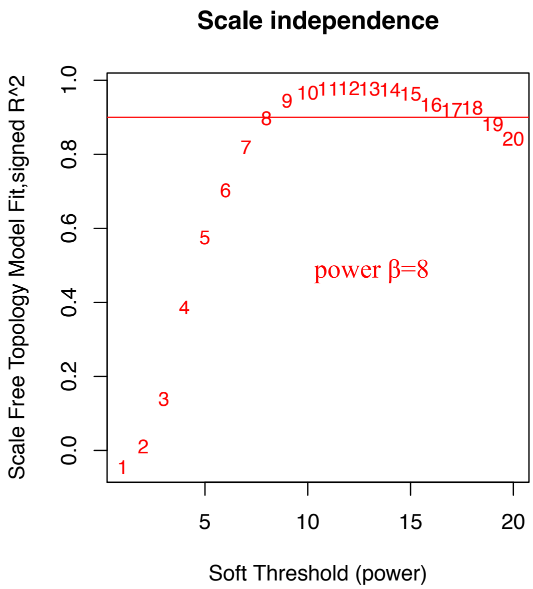

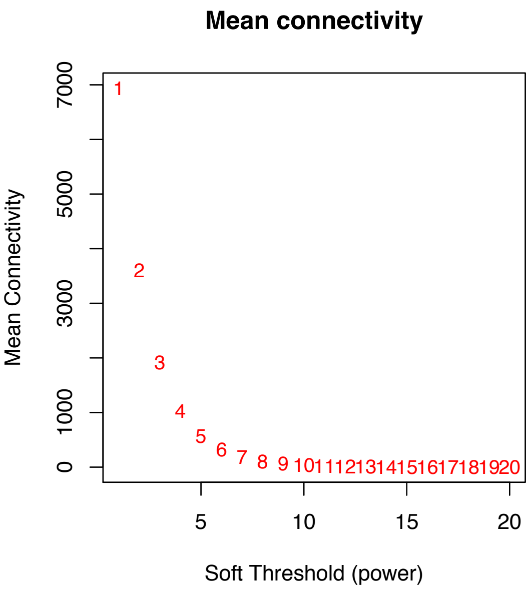

71 modules : identified

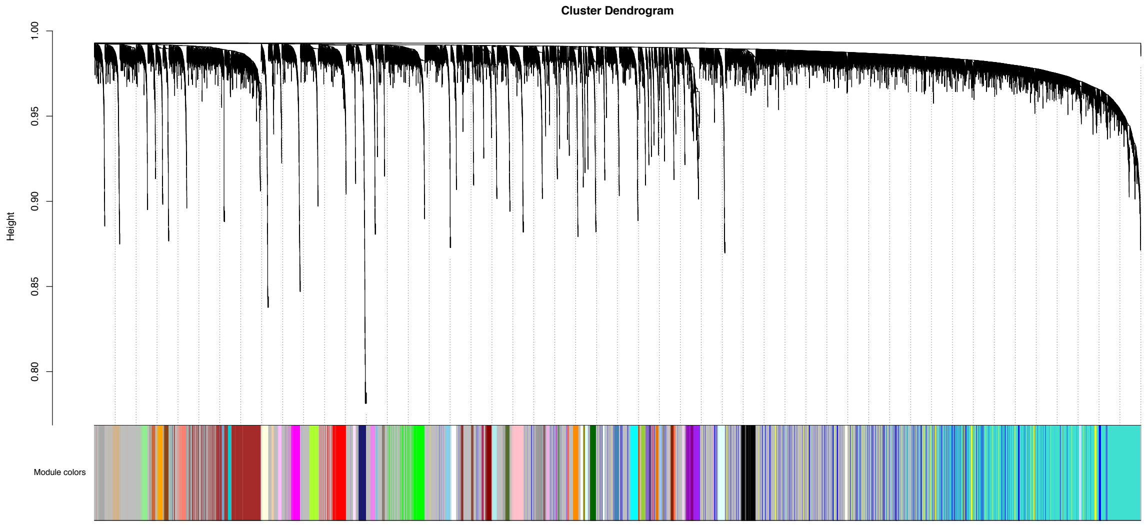

B

WGCNA network : II-18

micro-chamber dataset 22913 genes/ 94 cells
--> remove outlier cells
(s_265, s_281, s_282)
--> remove low expression genes
(5rpkm> at least 1 cell)
--> 13594 genes / 91 cells
(DMSO 47 cells, gefitinib 44 cells)

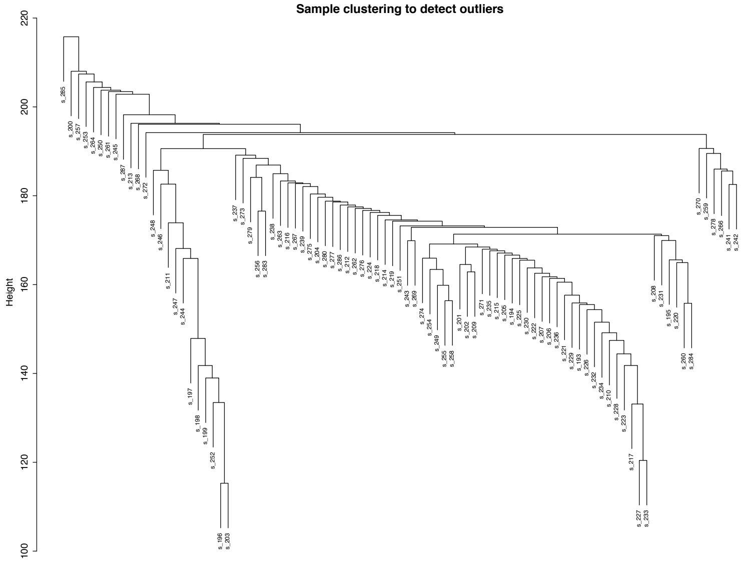

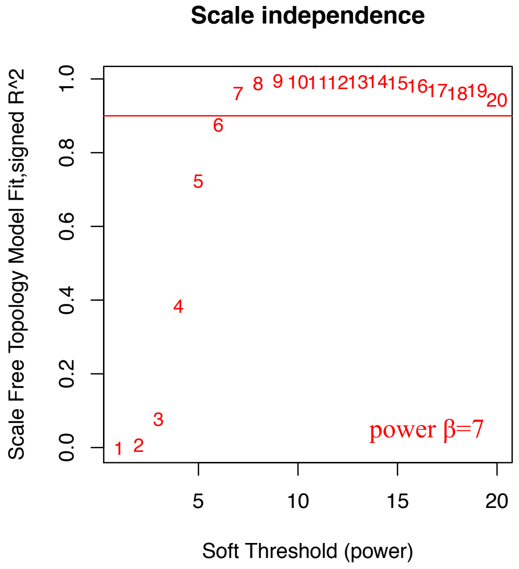

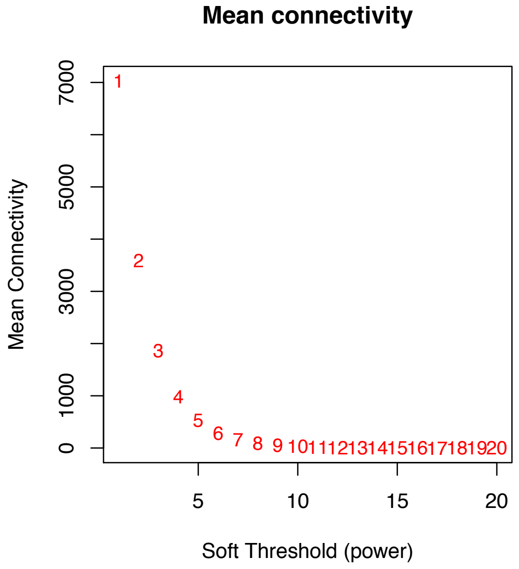

58 modules : identified

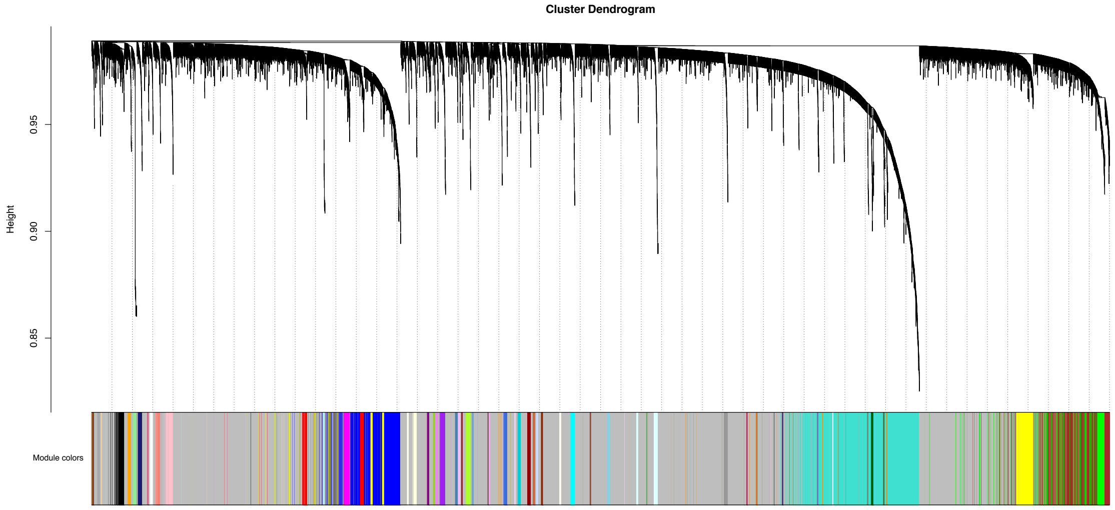

c

WGCNA network : H1650

micro-chamber dataset 22913 genes/ 94 cells
--> remove outlier cells
(s_331, s_333)
--> remove low expression genes
(5rpkm> at least 1 cell)
--> 14429 genes / 92 cells
(DMSO 45 cells, gefitinib 47 cells)

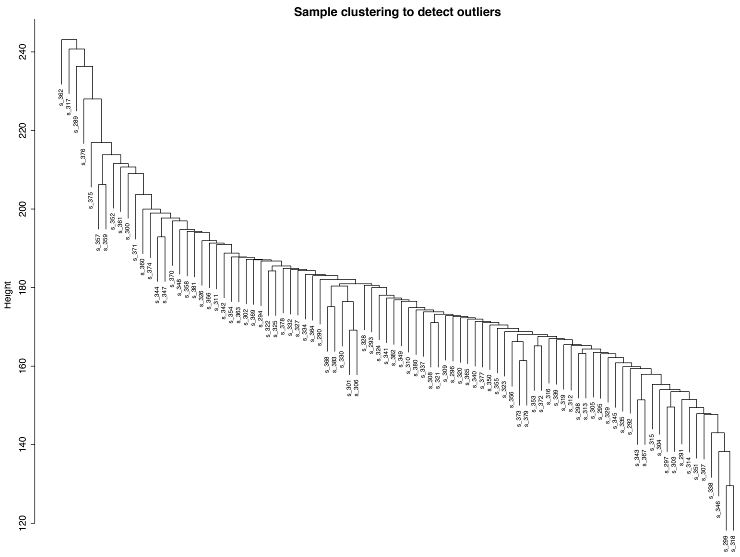

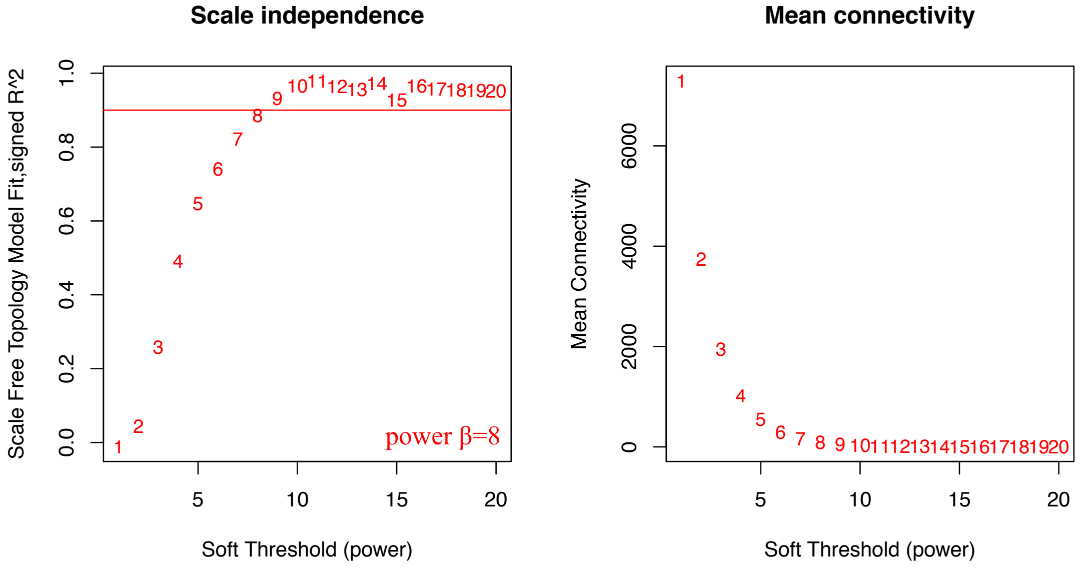

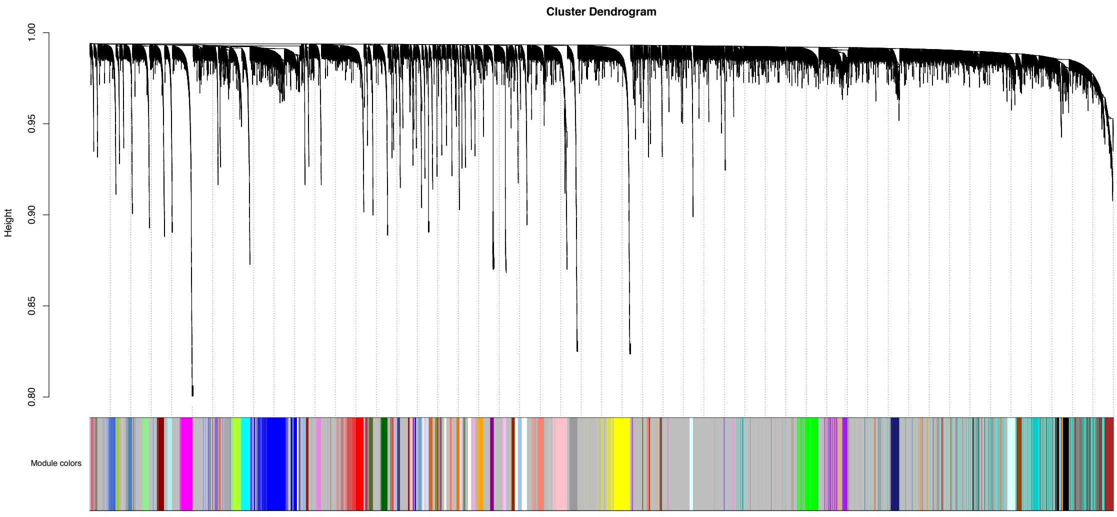

80 modules : identified

D

WGCNA network : H1975

micro-chamber dataset 22913 genes/ 94 cells
--> remove outlier cells
(s_145)
--> remove low expression genes
(5rpkm> at least 1 cell)
--> 14272 genes / 93 cells
(DMSO 47 cells, gefitinib 46 cells)

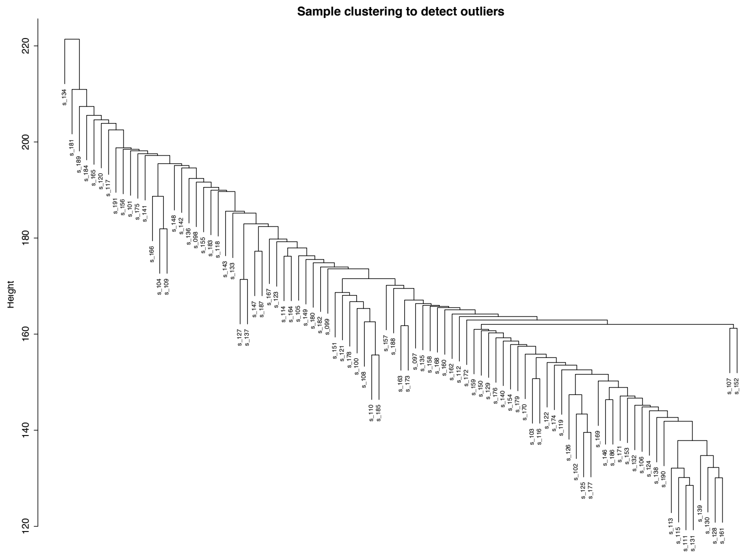

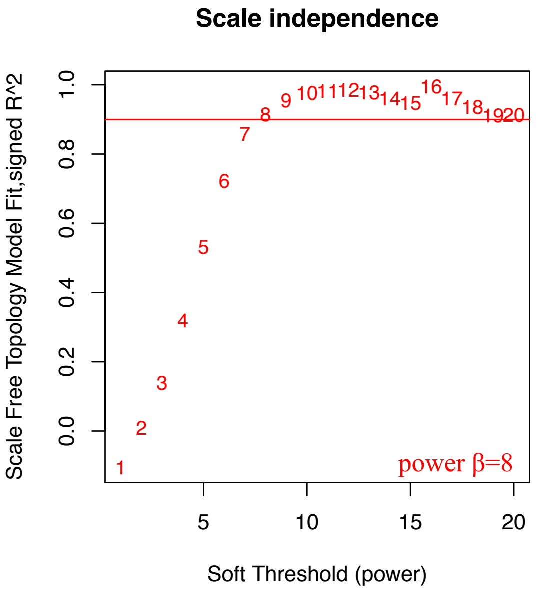

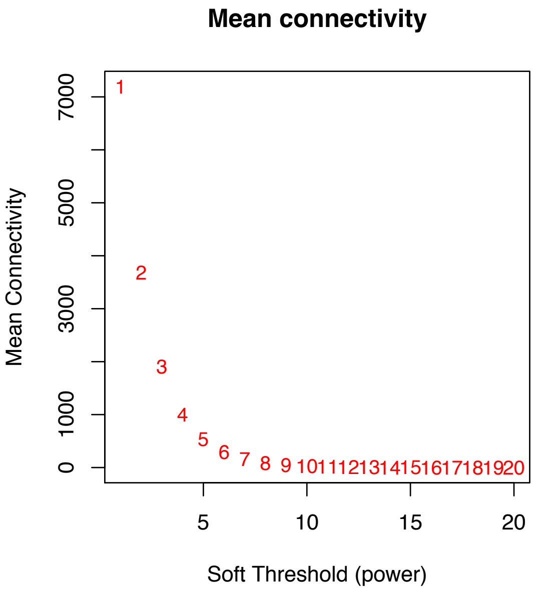

83 modules : identified

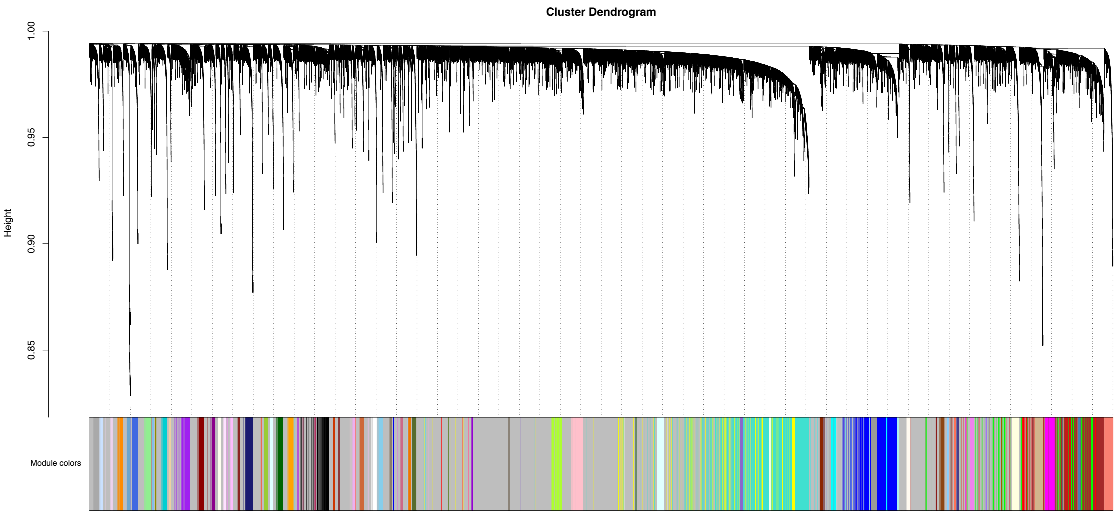

E

WGCNA network : H2228

micro-chamber dataset 22913 genes/ 94 cells
--> remove outlier cells
(s_428, s_458)
--> remove low expression genes
(5rpkm> at least 1 cell)
--> 14293 genes / 92 cells
(DMSO 47 cells, gefitinib 45 cells)

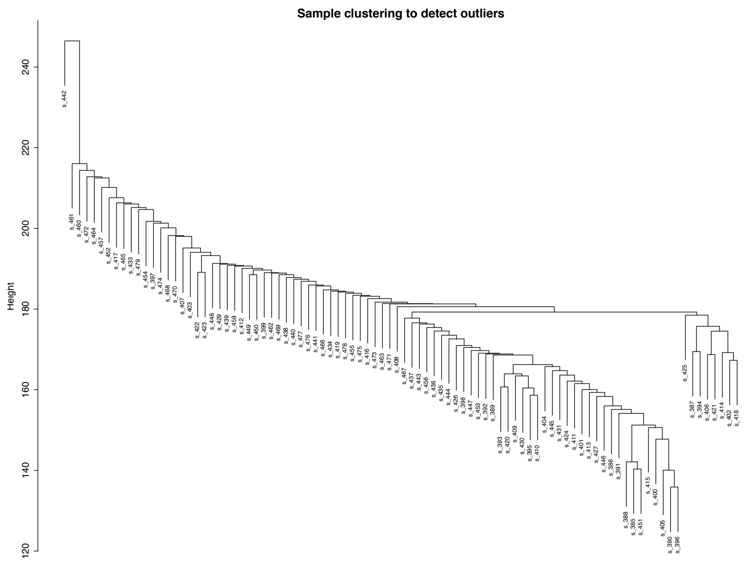

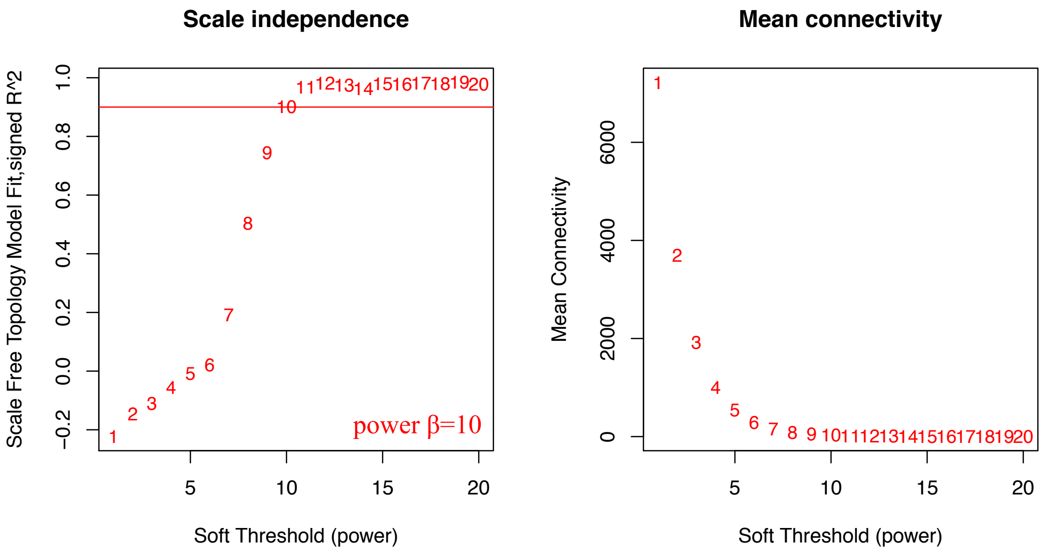

75 modules identified

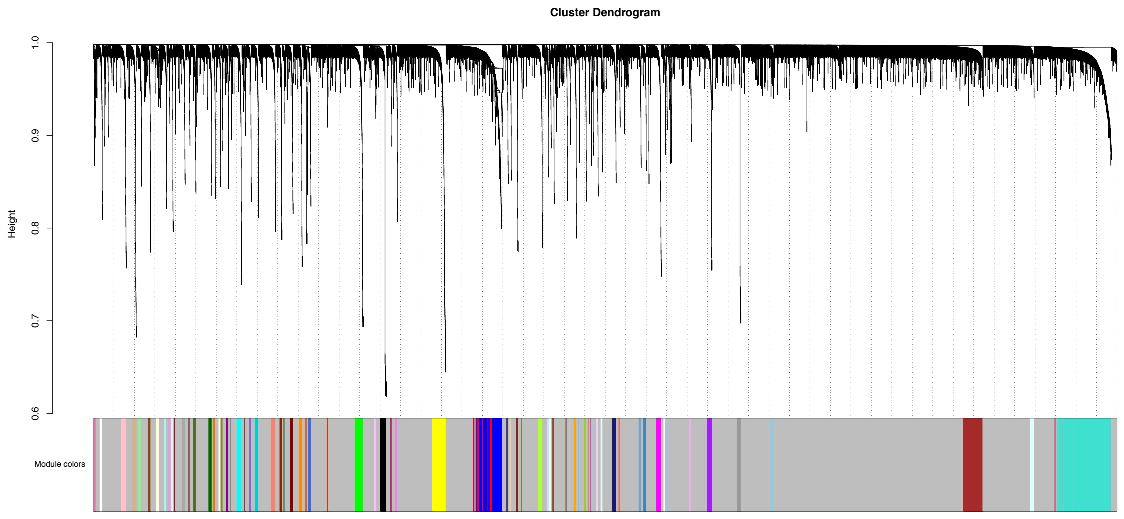

Supplemental Figure S6: Construction of network modules by WGCNA

(A-E) Schematic of the construction of the co-expression network with the micro-chamber dataset by WGCNA. The panel shows the schematics of the pre-process (left, top). On the top right, an analysis of network topology for powers is shown. The scale independence shows the scale-free fit index (*y*-axis) as a function of the soft-thresholding power (*x*-axis). The soft-thresholding power value used in the present study is shown in the plot as powerβ. The mean connectivity displays the mean connectivity (degree, *y*-axis) as a function of the soft-thresholding power (*x*-axis). The red line shown in left panel indicates a high value, 0.9. A clustering dendrogram of samples based on their Euclidean distance is shown on the bottom left. A clustering dendrogram of genes with dissimilarity based on topological overlap, together with assigned module colors, is shown on the bottom right. The number of identified modules is shown in the plot. Each figure shows the schematics of (A) PC9, (B) II-18, (C) H1650, (D) H1975 and (E) H2228.

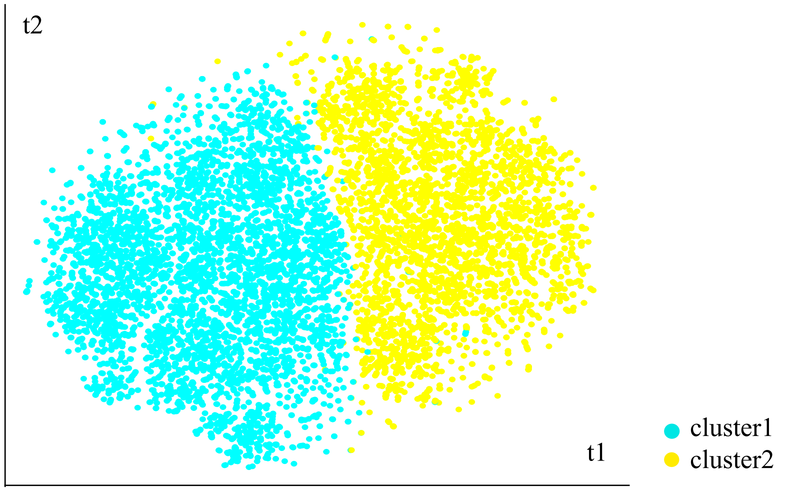

Supplemental Figure S7: Clustering of the micro-droplet datasets using module “PC9-magenta”

A

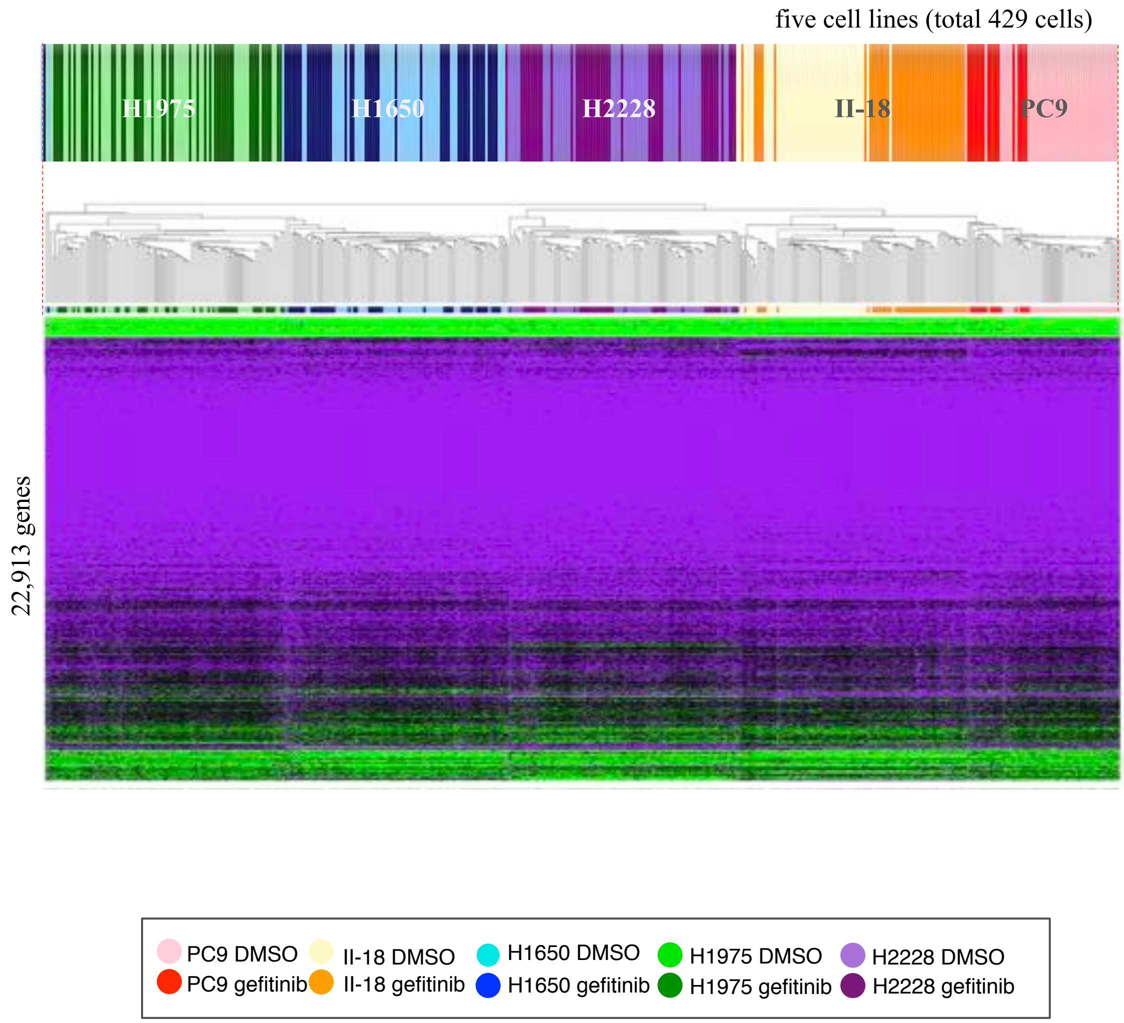

Supplemental Figure S8: Clustering of micro-chamber datasets

(A) Hierarchal clustering of 429 micro-chamber datasets by all 22,913 genes. All cell line datasets were gathered and clustered

B

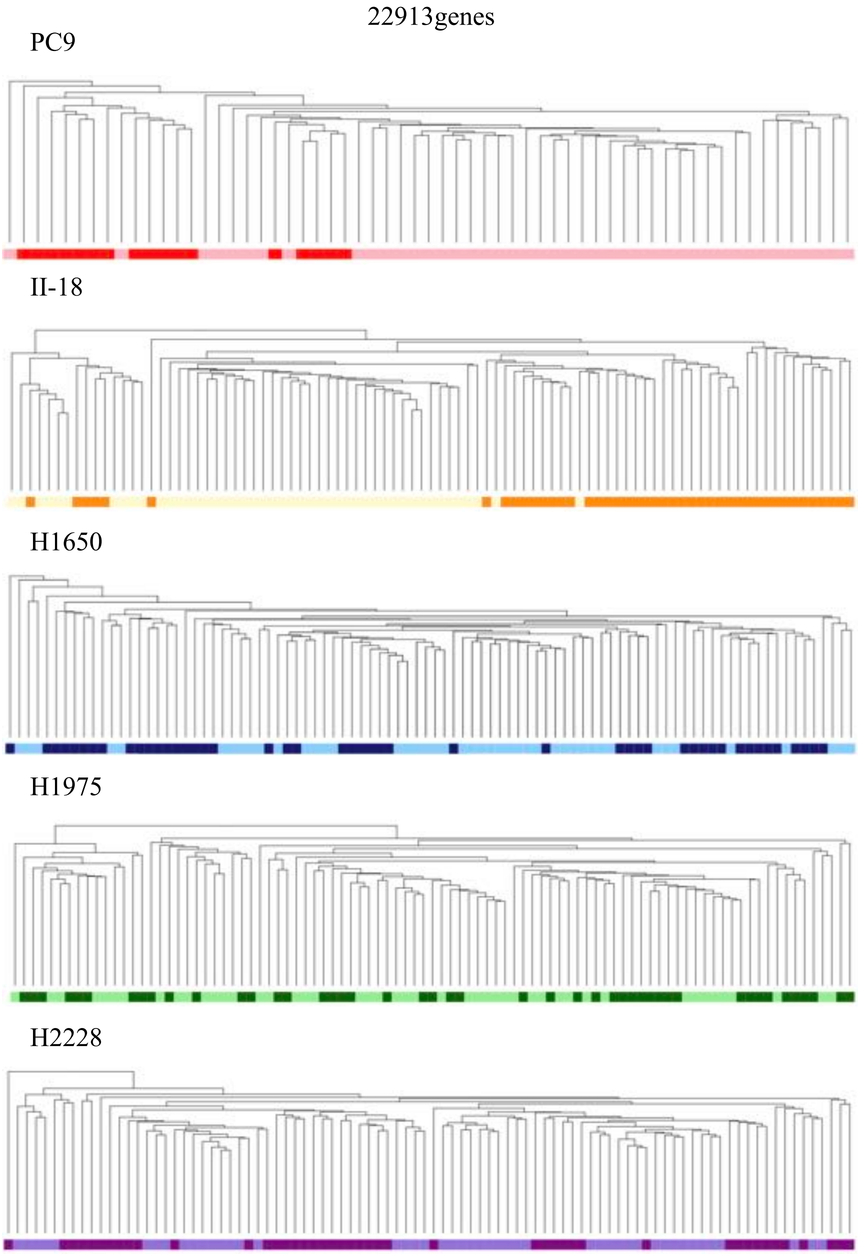

Supplemental Figure S8: Clustering of micro-chamber datasets
(B) Hierarchal clustering of 429 micro-chamber datasets by all 22,913 genes. Eeach cell line dataset was gathered and clustered.

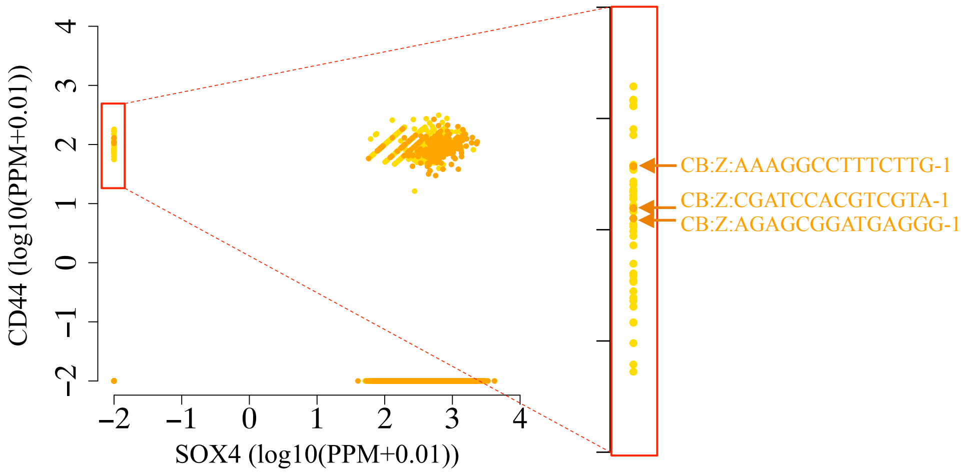

Supplemental Figure S9: Expression levels of SOX4 and CD44 in II-18 cells.
The plot shows the expression levels of SOX4 and CD44 in micro-droplet II-18 samples.

Supplemental Tables

Supplemental Table S1, Number and ratio without PCR sister reads in the micro-droplet dataset.

		Total raw reads	Reads without PCR sisters	%
PC9	DMSO	127631437	86320712	68
	gefitinib	170913768	73809216	43
II-18	DMSO	173605651	82652598	48
	gefitinib	164734523	98278555	60
H1650	DMSO	100094271	68329141	68
	gefitinib	111008829	76768723	69
H1975	DMSO	161777133	70871586	44
	gefitinib	173096952	115455843	67
H2228	DMSO	176666104	96259797	54
	gefitinib	149011834	67743954	45
		average	83649013	57

Supplemental Table S2, Gene list used in cell cycle analysis in micro-chamber datasets

Symbol	gene name
CCNE1	cyclin E1
E2F1	E2F transcription factor 1
CDC6	cell division cycle 6
PCNA	proliferating cell nuclear antigen
RFC4	replication factor C (activator 1) 4, 37kDa
DHFR	dihydrofolate reductase
RRM2	ribonucleotide reductase M2
RAD51	RAD51 recombinase
CDK1	cyclin-dependent kinase 1
TOP2A	topoisomerase (DNA) II alpha 170kDa
CCNF	cyclin F
CCNA2	cyclin A2
AURKA	aurora kinase A
BUB1	BUB1 mitotic checkpoint serine/threonine kinase
CCNB1	cyclin B1
PLK1	polo-like kinase 1
PTTG1	pituitary tumor-transforming 1
RAD21	RAD21 homolog (S. pombe)
VEGFC	vascular endothelial growth factor C
CDKN3	cyclin-dependent kinase inhibitor 3

Supplemental Table S3, Gene list used in cell cycle analysis in micro-droplet datasets

phase	Symbol	R
G1/S	ACD	0.2084462
G1/S	APEX2	0.2148424
G1/S	CCNE1	0.2663608
G1/S	CCNE2	0.3252323
G1/S	CDC6	0.3118351
G1/S	CDCA7L	0.2902344
G1/S	CHAF1A	0.2369656
G1/S	CLSPN	0.3776165
G1/S	E2F1	0.2836067
G1/S	GINS2	0.4020135
G1/S	GMNN	0.4269952
G1/S	INTS8	0.2561322
G1/S	LUC7L3	0.2050314
G1/S	MCM2	0.2240236
G1/S	MCM5	0.2868178
G1/S	MCM6	0.2503445
G1/S	NASP	0.3089949
G1/S	PCNA	0.5877051
G1/S	POLD3	0.2613784
G1/S	SLBP	0.5079985
G1/S	UNG	0.2762654
S	ASF1B	0.241012
S	CDC45	0.2327182
S	CENPM	0.2157942
S	ENOSF1	0.4931348
S	FEN1	0.2346011
S	GCLM	0.2522648
S	H1F0	0.2029316
S	HIST1H4C	0.36078
S	RRM2	0.3137284
S	RSRC2	0.2007431
S	TOP2A	0.2312718
S	TYMS	0.4957512
S	UBE2T	0.3587391
S	ZWINT	0.4421044

phase	Symbol	R
G2/M	ANLN	0.2723649
G2/M	AURKB	0.5321099
G2/M	BORA	0.2714558
G2/M	BUB3	0.2748968
G2/M	CCNA2	0.4965628
G2/M	CCNF	0.2104578
G2/M	CDCA2	0.2311073
G2/M	CDCA3	0.400363
G2/M	CDCA8	0.2783313
G2/M	CDK1	0.5821498
G2/M	CDKN2C	0.2262832
G2/M	CKAP2	0.3141243
G2/M	CKAP2L	0.2641564
G2/M	FAM83D	0.5195265
G2/M	GAS2L3	0.2245058
G2/M	H2AFX	0.2285597
G2/M	HJURP	0.4282288
G2/M	HMGB2	0.6292196
G2/M	KIF5B	0.3209594
G2/M	KIF20B	0.2873333
G2/M	KIF22	0.2844896
G2/M	KIF23	0.332756
G2/M	KIFC1	0.2672676
G2/M	KPNA2	0.6223326
G2/M	MAD2L1	0.2339825
G2/M	MALAT1	0.409941
G2/M	NDC80	0.3773631
G2/M	NUCKS1	0.2874382
G2/M	NUSAP1	0.4998711
G2/M	PIF1	0.2399757
G2/M	PSMD11	0.2376324
G2/M	PSRC1	0.3390074
G2/M	RCCD1	0.2086352
G2/M	SAP30	0.2066004
G2/M	SMC4	0.449287
G2/M	TOP2A	0.4822954
G2/M	TUBB	0.4278037
G2/M	TUBB4B	0.4368572
G2/M	UBE2C	0.6418814

phase	Symbol	R
M	AKIRIN2	0.2031315
M	ANLN	0.2958071
M	ANP32B	0.2541205
M	ANP32E	0.255035
M	ARL6IP1	0.628574
M	AURKA	0.6092834
M	BIRC5	0.3754414
M	BUB1	0.2367238
M	CCNA2	0.5522542
M	CCNB2	0.5141196
M	CDC20	0.6619391
M	CDC25B	0.2580322
M	CDCA3	0.4926722
M	CENPA	0.476688
M	CENPE	0.4081029
M	CENPF	0.5463907
M	CKAP2	0.3407493
M	CKS1B	0.2007793
M	CKS2	0.49405
M	DEPDC1	0.475011
M	DEPDC1B	0.2824315
M	DLGAP5	0.2684237
M	DNAJA1	0.2247345
M	FAM64A	0.4000655
M	GAS2L3	0.229844
M	GTSE1	0.3815512
M	HMGB3	0.3296062
M	HMMR	0.525004
M	HN1	0.3157104
M	HSPA8	0.4904216
M	KIF2C	0.310216
M	KIF5B	0.3977087
M	KIF20B	0.3202733
M	LBR	0.2530543
M	MKI67	0.3967232
M	MZT1	0.2896028
M	NEK2	0.4019897
M	NUF2	0.407485
M	NUSAP1	0.5026313
M	PBK	0.2675716
M	PLK1	0.6684096
M	PRR11	0.2214031
M	RBM8A	0.2013137
M	SFPQ	0.2199022
M	SGOL2	0.4225035
M	SRSF3	0.2975546
M	TACC3	0.2837015
M	THRAP3	0.2032257
M	TPX2	0.5248069
M	TTK	0.2213745
M	TUBB4B	0.5222375
M-G1	ANP32E	0.259491
M-G1	CDKN3	0.47832
M-G1	DKC1	0.2441299
M-G1	DYNLL1	0.3258742
M-G1	GRPEL1	0.2068026
M-G1	HSPA8	0.4251107
M-G1	ILF2	0.3342597
M-G1	KIF5B	0.2619027
M-G1	LYAR	0.2317684
M-G1	MORF4L2	0.3570747
M-G1	MRPS18B	0.2187978
M-G1	NUCKS1	0.3798808
M-G1	NUP37	0.2350708
M-G1	PBK	0.2099027
M-G1	PRC1	0.2334793
M-G1	PTTG1	0.6069561
M-G1	RAD21	0.258954
M-G1	RAN	0.4816124
M-G1	SRSF3	0.3415955

Supplemental Table S4, A list of the 28 genes included in the PC9 module "lightsteelblue1".

gene symbol	NCBI gene id
CENPA	NM_001042426,NM_001809
SGOL2	NM_001160033,NM_001160046,NM_152524
CKAP2L	NM_001304361,NM_152515
RAD21	NM_006265
CNTRL	NM_007018
STIL	NM_001048166,NM_001282936,NM_001282937,NM_001282938,NM_001282939,NM_003035
POLH	NM_001291969,NM_001291970,NM_006502
GPSM2	NM_013296
TTK	NM_001166691,NM_003318
NUF2	NM_031423,NM_145697
LARGE	NM_004737,NM_133642
HMMR	NM_001142556,NM_001142557,NM_012484,NM_012485
KIFC1	NM_002263
AURKA	NM_003600,NM_198433,NM_198434,NM_198435,NM_198436,NM_198437
BUB1	NM_001278616,NM_001278617,NM_004336
PIF1	NM_001286496,NM_001286497,NM_001286499,NM_025049
TROAP	NM_001100620,NM_001278324,NM_005480
TRAIP	NM_005879
IFNAR1	NM_000629
DEPDC1	NM_001114120,NM_017779
SPDL1	NM_017785
NCAPG	NM_022346
ERN2	NM_033266
KNSTRN	NM_001142761,NM_001142762,NM_033286
CKAP2	NM_001098525,NM_001286686,NM_001286687,NM_018204
FAM72B	NM_001100910
CENPE	NM_001286734,NM_001813
HACD4	NM_001010915
KIF14	NM_014875
CDC25C	NM_001287582,NM_001287583,NM_001790,NM_022809
CDCA2	NM_152562
RNF26	NM_032015
PSRC1	NM_001005290,NM_001032291,NM_032636
GAS2L3	NM_001303130,NM_001303131,NM_174942
KIF15	NM_020242
ESPL1	NM_012291
AURKAPS1	NR_001587
HMMR-AS1	NR_109892

Supplemental Table S5, Result of the gene ontology term enrichment analysis of the module “lightsteelblue1”.

rank	enrichmentP	BonferoniP	termID	termOntology	termName
1	6.42E-16	9.45E-12	GO:0022402	BP	cell cycle process
2	4.05E-15	5.95E-11	GO:0000278	BP	mitotic cell cycle
3	4.81E-15	7.08E-11	GO:0007067	BP	mitotic nuclear division
4	5.98E-15	8.79E-11	GO:0007059	BP	chromosome segregation
5	6.21E-15	9.13E-11	GO:0051301	BP	cell division
6	2.80E-13	4.12E-09	GO:0007049	BP	cell cycle
7	4.95E-12	7.28E-08	GO:0007017	BP	microtubule-based process
8	7.64E-12	1.12E-07	GO:0000819	BP	sister chromatid segregation
9	4.02E-11	5.91E-07	GO:0005819	CC	spindle
10	1.68E-10	2.47E-06	GO:0015630	CC	microtubule cytoskeleton

Supplemental Table S6, A list of the 125 genes included in the PC9 module "magenta".

Gene Symbol	NCBI gene id
COG3	NM_031431
ZNF845	NM_138374
PLD1	NM_001130081,NM_002662
DUSP1	NM_004417
RGCC	NM_014059
TRIM52	NM_032765
HMOX1	NM_002133
PITX2	NM_000325,NM_001204397,NM_001204398,NM_001204399,NM_153426,NM_153427
GDPD1	NM_001165993,NM_001165994,NM_182569
RNF2	NM_007212
WDR78	NM_024763,NM_207014
JMY	NM_152405
ELF1	NM_001145353,NM_172373
NAV2	NM_001111018,NM_001111019,NM_001244963,NM_145117,NM_182964
ASAP3	NM_001143778,NM_017707
ASB7	NM_024708,NM_198243
ATXN7	NM_000333,NM_001128149,NM_001177387
PSG8	NM_001130167,NM_001130168,NM_182707
PARP16	NM_017851
ACTG1	NM_001199954,NM_001614
PRR34	NM_018280
SNAI2	NM_003068
C19orf73	NM_018111
CCDC153	NM_001145018
TFEB	NM_001167827,NM_001271943,NM_001271944,NM_001271945,NM_007162
GSDMB	NM_001042471,NM_001165958,NM_001165959,NM_018530
GTDC1	NM_001006636,NM_001164629,NM_001284233,NM_001284234,NM_001284235,NM_001284238,NM_024659
TMEM14C	NM_001165258,NM_016462
RAB42	NM_001193532,NM_152304
THBD	NM_000361
KPNA5	NM_002269
PLCE1	NM_001165979,NM_001288989,NM_016341
CFHR1	NM_002113
KBTBD6	NM_152903
KRTAP2-1	NM_001123387
OSTM1	NM_014028
CSRP3	NM_003476
HDAC10	NM_001159286,NM_032019
SYTL3	NM_001009991,NM_001242384,NM_001242394,NM_001242395
ATF3	NM_001030287,NM_001040619,NM_001206484,NM_001206486,NM_001206488,NM_001674
ARHGEF37	NM_001001669
ANO8	NM_020959
HIST1H2AI	NM_003509
CFP	NM_001145252,NM_002621
EPM2AIP1	NM_014805
SAMD4A	NM_001161576,NM_001161577,NM_015589
KRTAP2-4	NM_033184
KRTAP2-2	NM_033032
TSSK2	NM_053006
PTGER4	NM_000958
CCNT1	NM_001240,NM_001277842
FAM209A	NM_001012971
SGK1	NM_001143676,NM_001143677,NM_001143678,NM_001291995,NM_005627
CCDC18	NM_206886
GAS1	NM_002048
TDRP	NM_001256113,NM_175075
RND3	NM_001254738,NM_005168
RHOB	NM_004040
KRTAP2-3	NM_001165252
CLEC2B	NM_005127
SLC22A20	NM_001004326
SPTBN2	NM_006946
TNFRSF10D	NM_003840
CDK18	NM_002596,NM_212502,NM_212503
NFIL3	NM_001289999,NM_001290000,NM_005384
HIST1H2AJ	NM_021066
TULP2	NM_003323
SBNO2	NM_001100122,NM_014963
ID4	NM_001546
VAMP4	NM_001185127,NM_003762
TMEM65	NM_194291
TRPV4	NM_001177428,NM_001177431,NM_001177433,NM_021625,NM_147204
DCUN1D4	NM_001040402,NM_001287755,NM_001287757,NM_015115
WDSUB1	NM_001128212,NM_001128213,NM_152528
PUS10	NM_144709
AOC2	NM_001158,NM_009590
PKP1	NM_000299,NM_001005337
IL6	NM_000600
LRRC69	NM_001129890
SLC2A10	NM_030777
COL17A1	NM_000494
AMIGO3	NM_198722
IFNE	NM_176891
ATP6AP1L	NM_001017971
RARA	NM_000964,NM_001024809,NM_001145301,NM_001145302
MYLIP	NM_013262
RNF38	NM_022781,NM_194328,NM_194329,NM_194330,NM_194332
ANKRD65	NM_001145210,NM_001243535,NM_001243536
SCRN3	NM_001193528,NM_024583
GLP2R	NM_004246
SOCS6	NM_004232
AQP11	NM_173039
FBXO32	NM_001242463,NM_058229,NM_148177
IRGQ	NM_001007561
MAST4	NM_001164664,NM_001290226,NM_001290227,NM_001290228,NM_001297651,NM_015183,NM_198828
IGIP	NM_001007189
ACAD10	NM_001136538,NM_025247
ZNF248	NM_001267597,NM_001267605,NM_001267606,NM_001267607,NM_021045
GPR25	NM_005298
ZFP36	NM_003407
TRAPPC8	NM_014939
UPRT	NM_145052
CELSR2	NM_001408
FOXJ2	NM_018416
TMEM81	NM_203376
LOC100130331	NR_027247
ZNF197-AS1	NR_046658
LOC155060	NR_036573
LINC00511	NR_033876
LOC100126784	NR_015384
LOC101927571	NR_110782
MIR6792	NR_106850
DKFZP434I0714	NR_033797
LOC283440	NR_033958
PPP1R26-AS1	NR_038969
LOC100379224	NR_033341
PAN3-AS1	NR_029383
LOC101927267	NR_120449
PIK3IP1-AS1	NR_110542
LINC01006	NR_103858
MIR6880	NR_106940
MIR1231	NR_031599
LOC102724000	NR_121615
ACTG1P20	NR_033926
AGBL5-AS1	NR_046730

Supplemental Table S7, Result of the gene ontology term enrichment analysis of the module "magenta".

rank	enrichmentP	BonferoniP	termID	termOntology	termName
1	9.01E-06	0.132589083	GO:0006959	BP	humoral immune response
2	0.00013173	1	GO:0002366	BP	leukocyte activation involved in immune response
3	0.000143901	1	GO:0002286	BP	T cell activation involved in immune response
4	0.000225331	1	GO:0050710	BP	negative regulation of cytokine secretion
5	0.000393841	1	GO:0045630	BP	positive regulation of T-helper 2 cell differentiation
6	0.000445651	1	GO:0001078	MF	RNA polymerase II core promoter proximal region sequence-specific DNA binding transcription factor activity involved in negative regulation of transcription
7	0.000463626	1	GO:0000982	MF	RNA polymerase II core promoter proximal region sequence-specific DNA binding transcription factor activity
8	0.000529063	1	GO:0008081	MF	phosphoric diester hydrolase activity
9	0.000548049	1	GO:0006956	BP	complement activation
10	0.000548049	1	GO:0042093	BP	T-helper cell differentiation

Supplemental Table S8, A gene list of the module II-18 "red"

Gene symbol	NCBI gene id
CCNO	NM_021147
AKR1A1	NM_001202413,NM_001202414,NM_006066,NM_153326
ATP6V0E1	NM_003945
CDKN2B	NM_004936,NM_078487
ASAH1	NM_001127505,NM_004315,NM_177924
CSF1	NM_000757,NM_172210,NM_172211,NM_172212
SOX4	NM_003107
HIST2H2BE	NM_003528
ALG1L	NM_001015050,NM_001195223
DSTN	NM_001011546,NM_006870
HLA-A	NM_001242758,NM_002116
BMF	NM_001003940,NM_001003942,NM_001003943,NM_033503
HIST1H2BD	NM_021063,NM_138720
TEAD2	NM_001256658,NM_001256659,NM_001256660,NM_001256661,NM_001256662,NM_003598
ANXA4	NM_001153
GOLGA2	NM_004486
MXD4	NM_006454
TP53I11	NM_001258320,NM_001258321,NM_001258322,NM_001258323,NM_001258324,NM_006034
BRD2	NM_001113182,NM_001199455,NM_001199456,NM_001291986,NM_005104
CERS4	NM_024552
HLA-C	NM_001243042,NM_002117
KDR	NM_002253
B2M	NM_004048
CORO1B	NM_001018070,NM_020441
CETN2	NM_004344
MSMO1	NM_001017369,NM_006745
EFNA1	NM_004428,NM_182685
CEACAM3	NM_001277163,NM_001815
BPHL	NM_001302777,NM_004332
ATP6V1G1	NM_004888
EIF2AK1	NM_001134335,NM_014413
PCGF3	NM_006315
SMIM14	NM_174921
SRSF8	NM_032102
TMEM150A	NM_001031738
TMEM219	NM_001083613,NM_194280
ICA1	NM_001136020,NM_001276478,NM_004968,NM_022307
CLSTN3	NM_014718
ERGIC3	NM_015966,NM_198398
ITM2B	NM_021999
TMEM230	NM_001009923,NM_001009924,NM_001009925,NM_014145
TMEM263	NM_152261
YIPF3	NM_015388
TMEM59	NM_004872
SEC22B	NM_004892
CLDN4	NM_001305
WBP1	NM_012477
ARF4	NM_001660
FAM114A1	NM_138389
CEACAM5	NM_001291484,NM_004363
LSS	NM_001001438,NM_001145436,NM_001145437,NM_002340
CHMP1B	NM_020412
FDFT1	NM_001287742,NM_001287743,NM_001287744,NM_001287745,NM_001287747,NM_001287748,NM_001287749,NM_001287750,NM_001287751,NM_001287756,NM_004462
SDPR	NM_004657
GSTA4	NM_001512
PERP	NM_022121
SLC5A2	NM_003041
GPR110	NM_025048,NM_153840
HLA-B	NM_005514
ERV3-1	NM_001007253
SND1	NM_014390
CCS	NM_005125
RNF144B	NM_182757
CCNG2	NM_004354

Gene symbol	NCBI gene id
PLEKHB1	NM_001130033,NM_001130034,NM_001130035,NM_001130036,NM_021200
HLA-F	NM_001098478,NM_001098479,NM_018950
SRI	NM_001256891,NM_001256892,NM_003130,NM_198901
TMED9	NM_017510
PDCD4	NM_001199492,NM_014456,NM_145341
SC5D	NM_001024956,NM_006918
BET1	NM_005868
GOLGB1	NM_001256486,NM_001256487,NM_001256488,NM_004487
TTC3	NM_001001894,NM_003316
OAZ2	NM_001301302,NM_002537
ELF3	NM_001114309,NM_004433
SCARB2	NM_001204255,NM_005506
IFT57	NM_018010
ST13	NM_001278589,NM_003932
LRIG1	NM_015541
ATP6AP1	NM_001183
ALDH3B1	NM_000694,NM_001030010,NM_001161473,NM_001290058,NM_001290059
PELI1	NM_020651
CREB3	NM_006368
SQLE	NM_003129
COPA	NM_001098398,NM_004371
MIR205HG	NM_001104548
NICN1	NM_032316
CBLB	NM_170662
UBE2R2	NM_017811
MAGED1	NM_001005332,NM_001005333,NM_006986
STEAP4	NM_001205315,NM_001205316,NM_024636
KDELR1	NM_006801
WIPI2	NM_001033518,NM_001033519,NM_001033520,NM_001278299,NM_015610,NM_016003
HMGCS1	NM_001098272,NM_002130
INSIG1	NM_005542,NM_198336,NM_198337
HLA-G	NM_002127
GNAL	NM_001142339,NM_001261443,NM_001261444,NM_182978
NARF	NM_001038618,NM_001083608,NM_012336,NM_031968
SCD	NM_005063
MOSPD3	NM_001040097,NM_001040098,NM_001040099,NM_023948
KLHL24	NM_017644
SMPDL3B	NM_001009568,NM_001304579,NM_014474
TKT	NM_001064,NM_001135055,NM_001258028
HBP1	NM_001244262,NM_012257
CAPN1	NM_001198868,NM_001198869,NM_005186
UBXN1	NM_001286077,NM_001286078,NM_015853
PBXIP1	NM_020524
DPP3	NM_001256670,NM_005700,NM_130443
DNAJC4	NM_005528
YPEL5	NM_001127399,NM_001127400,NM_001127401,NM_016061
TMPRSS2	NM_001135099,NM_005656
SEC24C	NM_004922,NM_198597
PARP14	NM_017554
PPAPDC1B	NM_001102559,NM_001102560,NM_032483
LITAF	NM_001136472,NM_001136473,NM_004862
RPL4	NM_000968
ST8SIA4	NM_005668,NM_175052
C16orf58	NM_022744
NIPSNAP1	NM_001202502,NM_003634
PON2	NM_000305,NM_001018161
LOC102723885	NR_120517
LOC101927043	NR_110207,NR_110208
CIRBP-AS1	NR_027271
ZNF528-AS1	NR_125345
MIR4800	NR_039964
LOC440173	NR_027471
HLA-J	NR_024240
HLA-H	NR_001434
ST13P4	NR_002183
UGDH-AS1	NR_047679
ZNRD1-AS1	NR_026751
INO80B-WBP1	NR_037849

Supplemental Table S9, Number of cells used in WGCNA and hierarchal clustering

	DMSO	gefitinib
PC9	44	17
II-18	47	44
H1650	45	47
H1975	47	46
H2228	47	45
total	230	199
